# Efficacy and safety of pyrimidine nucleos(t)ide therapy in thymidine kinase 2 deficiency

**DOI:** 10.1093/braincomms/fcag201

**Published:** 2026-06-03

**Authors:** Michio Hirano, Caterina Garone, Richard Haas, Carmen Paradas, Fernando Scaglia, Irene Rebollo Mesa, Carl Chiang, Anny-Odile Colson, Susan VanMeter, Cristina Domínguez-González

**Affiliations:** Department of Neurology, H. Houston Merritt Center for Neuromuscular Disorders, Columbia University Irving Medical Center, New York, NY 10032, USA; Department of Medical and Surgical Sciences, Alma Mater Studiorum, University of Bologna, Bologna 40138, Italy; IRCCS Istituto delle Scienze Neurologiche, UOC Neuropsichiatria dell'età Pediatrica di Bologna, Bologna 40139, Italy; Department of Neurosciences, University of California San Diego and Rady Children’s Hospital, San Diego, CA 92093, USA; Neurology Department, Neuromuscular Disorders Unit, Instituto de Biomedicina de Sevilla, Hospital Universitario Virgen del Rocío, Consejo Superior de Investigaciones Científicas, University of Seville, Seville 41013, Spain; Center for Biomedical Network Research on Neurodegenerative Diseases (CIBERNED), Instituto de Salud Carlos III, Madrid 28029, Spain; Department of Molecular and Human Genetics, Baylor College of Medicine, Houston, TX 77030, USA; Texas Children’s Hospital, Houston, TX 77030, USA; Joint BCM-CUHK Center of Medical Genetics, Prince of Wales Hospital, Shatin, Hong Kong SAR 999077, China; Biometrics and Data Science, UCB, Madrid 28020, Spain; Global Clinical Development, UCB, Morrisville, NC 27560, USA; Safety Risk Management, UCB, Colombes, Paris 92700, France; Global Clinical Development, UCB, Morrisville, NC 27560, USA; Neuromuscular Unit, Neurology Department, Hospital Universitario 12 de Octubre, Madrid 28041, Spain; Mitochondrial and Neuromuscular Research Group '12 de Octubre', imas12 Research Institute, Madrid 28041, Spain; Biomedical Network Research Centre on Rare Diseases (CIBERER), Instituto de Salud Carlos III, Madrid 28029, Spain

**Keywords:** mitochondrial myopathy, pyrimidine nucleos(t)ide therapy, survival, thymidine kinase 2 deficiency, treatment efficacy

## Abstract

Thymidine kinase 2 deficiency (TK2d) (MIM 609560) is an ultra-rare, autosomal recessive mitochondrial myopathy caused by *TK2* variants, leading to mitochondrial DNA depletion and/or multiple deletions. People with thymidine kinase 2 deficiency experience progressive myopathy, bulbar weakness and respiratory insufficiency, often losing the ability to walk, eat and breathe independently. Doxecitine and doxribtimine represents the first approved treatment for patients with thymidine kinase 2 deficiency with age of symptom onset ≤12 years by the US Food and Drug Administration and the European Medicines Agency; previously, disease management was limited to supportive care. We investigated the efficacy and safety of pyrimidine nucleos(t)ide therapy in thymidine kinase 2 deficiency. Patients treated with pyrimidine nucleos(t)ides were pooled from retrospective (NCT03701568, NCT05017818) and prospective (NCT03845712) studies and company-supported Expanded Access Programs. Untreated patients were pooled from literature reviews and a retrospective chart review study (NCT05017818). Patient subgroups were stratified by age of thymidine kinase 2 deficiency symptom onset (≤12 years and >12 years). The primary outcome was survival in 50th-percentile matched pairs of treated and untreated patients. Other outcomes included status of developmental motor milestones, ventilatory and feeding tube support, and safety. In total, 218 patients were included (treated: 104; untreated: 114). Baseline demographics and characteristics were comparable between subgroups. Most patients had an age of symptom onset ≤12 years [treated: 82/104 (78.8%); untreated: 93/114 (81.6%)]. In the age-of-symptom-onset-≤12-years subgroup, restricted mean survival time (95% confidence interval) was 29.2 (28.2, 30.3) years over the 30 years after symptom onset for treated patients and 14.4 (11.1, 17.6) years for untreated patients. Loss of ≥1 acquired motor milestone was more frequent before treatment start than after. Substantially more patients regained ≥1 lost motor milestone after treatment start than before. Ventilatory and feeding support were used across all age-of-symptom-onset subgroups, but some patients reduced or discontinued support after starting treatment and fewer patients initiated support after treatment start than before. Most treatment-emergent adverse events (TEAEs) did not lead to discontinuation. The most frequent TEAE was diarrhoea [43/50 patients (86.0%)], which was generally mild or moderate and resolved with dose reduction. Serious TEAEs occurred in 28/50 patients (56.0%); few were considered to be drug related [4/50 (8.0%)]. In total, 3/67 patients (4.5%) experienced a fatal serious TEAE, which were not considered to be drug related. These findings indicate that pyrimidine nucleos(t)ide therapy improves survival and functional outcomes in people with thymidine kinase 2 deficiency, especially those with age of symptom onset ≤12 years, and has an acceptable safety profile.

## Teaser

Hirano *et al*. report on the efficacy and safety of pyrimidine nucleos(t)ide therapy in patients with thymidine kinase 2 deficiency, an ultra-rare genetic disease. The treatment was well tolerated and improved survival and movement abilities, especially in patients who developed symptoms at or before 12 years of age.

## Graphical Abstract

**Graphical Abstract:**
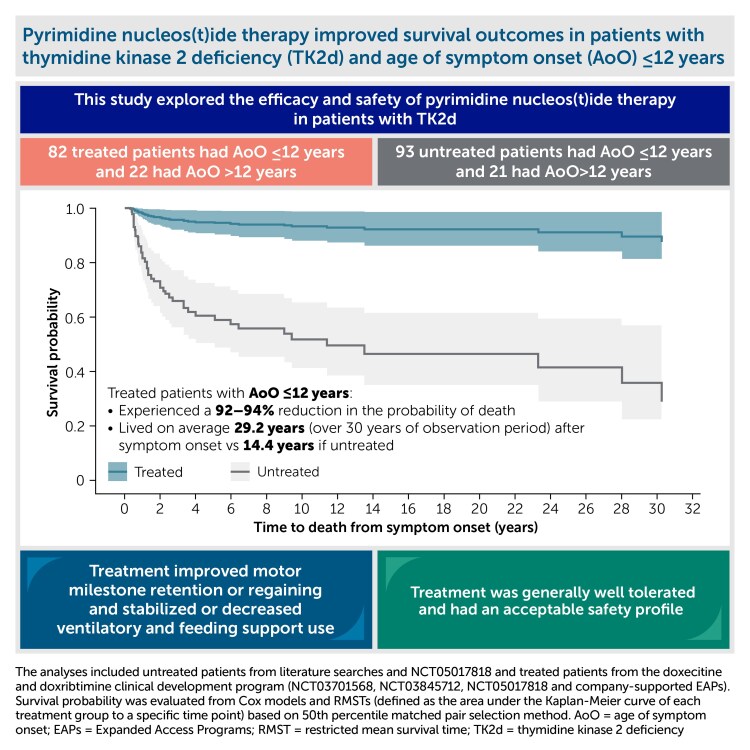

## Introduction

Thymidine kinase 2 deficiency (TK2d) (MIM 609560) is an ultra-rare, progressive, debilitating and often life-threatening autosomal recessive mitochondrial myopathy associated with high mortality and significant morbidity.^1-4^ Biallelic pathogenic variants of the thymidine kinase 2 (*TK2*) gene lead to impaired activity of the mitochondrial matrix enzyme thymidine kinase 2 (TK2), which is responsible for phosphorylating deoxycytidine (dC) and deoxythymidine (dT) to their respective deoxynucleoside monophosphates. These monophosphates are further phosphorylated to generate deoxynucleoside triphosphates, which are incorporated into replicating mitochondrial DNA (mtDNA).^3^ Impaired TK2 activity in TK2d results in an imbalance of the deoxynucleoside triphosphates pool, with mtDNA depletion and/or multiple mtDNA deletions leading to deficiencies in activities of mitochondrial respiratory chain complexes required for cellular energy production.^5,6^

Estimates suggest a TK2d prevalence of 1.64 people per million worldwide [first and third quartiles (Q1, Q3): 0.5, 3.1 per million].^7^ People with TK2d manifest progressive proximal and bulbar myopathy and respiratory weakness, and many lose the ability to walk, eat and breathe independently.^4,8-11^ Although isolated myopathy with early respiratory involvement is the most frequent presentation, TK2d may also affect other organ systems, including the CNS (seizures, encephalopathy, cognitive impairment), heart (cardiomyopathy, ventricular hypertrophy, arrhythmias), liver (liver dysfunction/failure) and kidneys (nephropathy).^3,4^ Respiratory failure due to respiratory muscle weakness is the primary cause of early death.^4,8-11^

TK2d manifests as a continuous phenotypic spectrum, however it has been suggested, based on clinical experience, that stratifying by age of symptom onset may be useful when describing the clinical forms of the disease.^3,4^ Some variability exists in the cut-offs used to define earlier-onset forms of TK2d (e.g. ≤1, ≤2 or ≤4 years); however, the cut-off of age of symptom onset >12 years has been largely agreed as clinically meaningful in describing the difference in disease presentation in terms of speed of progression, severity of outcomes and likely pathophysiology.^2-4^ People with TK2d and age of symptom onset ≤2 years tend to experience rapid progression to early death.^2-4^ Those with age of symptom onset >2 to ≤12 years generally experience slower progression to early death than those with age of symptom onset ≤2 years and, in most cases, loss of ambulation within 10 years and progression to use of ventilatory support.^2-4^ Those with age of symptom onset >12 years tend to experience the slowest progression compared with the other age-of-symptom-onset subgroups, with people retaining the ability to walk but often requiring ventilatory support.^2-4^ However, natural history data in the latter group of patients is limited, making disease trajectory and prognosis unclear.

As of November 2025 and March 2026, doxecitine and doxribtimine, an oral pyrimidine nucleoside therapy containing dC and dT, is the first approved treatment for paediatric and adult patients with TK2d with an age of symptom onset ≤12 years by the US Food and Drug Administration (FDA) and the European Medicines Agency (EMA), respectively.^12-14^ Prior to this approval, management of TK2d focused on supportive care, which does not change the progressive disease trajectory.^15^ People with TK2d are often managed by a multidisciplinary healthcare team that can include neurologists, clinical geneticists, physical therapists, pulmonologists, cardiologists and gastroenterologists. People with TK2d often rely on devices to assist with daily living, such as wheelchairs, feeding tubes and ventilators (invasive and non-invasive).^4^

Doxecitine and doxribtimine targets the underlying TK2d disease pathology by increasing substrate levels available for residual mitochondrial TK2 activity, as well as for thymidine kinase 1 and dC kinase activities in the cytosol.^16,17^ Results from MT-1621-101 (NCT03701568), a retrospective study of 38 patients with TK2d receiving dC and dT or their monophosphates at ≤800 mg/kg/day, strongly suggested that the therapy may reduce risk of death, improve attainment and/or retention of developmental motor milestones and help to stabilize disease progression in people with TK2d.^12^

Here, we build on findings from MT-1621-101 to report the Integrated Summary of Efficacy (ISE) and Integrated Summary of Safety (ISS) of treatment with pyrimidine nucleos(t)ides, using data pooled from MT-1621-101 and another retrospective study [MT-1621-107 (NCT05017818)], a prospective study [TK0102 (NCT03845712)] and company-supported Expanded Access Programs (EAPs), which together comprise the clinical development programme for use of doxecitine and doxribtimine in TK2d.

## Materials and methods

### Study design

The efficacy (ISE) and safety (ISS) analyses aimed to characterize effect of doxecitine and doxribtimine in patients with TK2d at a dose of ≤800 mg/kg/day (≤400 mg/kg/day each of doxecitine and doxribtimine). The source studies for the efficacy and safety populations are detailed in Supplementary Table 1, and study designs are presented in Supplementary Fig. 1; the methodology for MT-1621-101 has been published previously.^12^

### Patient population

**Figure 1:**
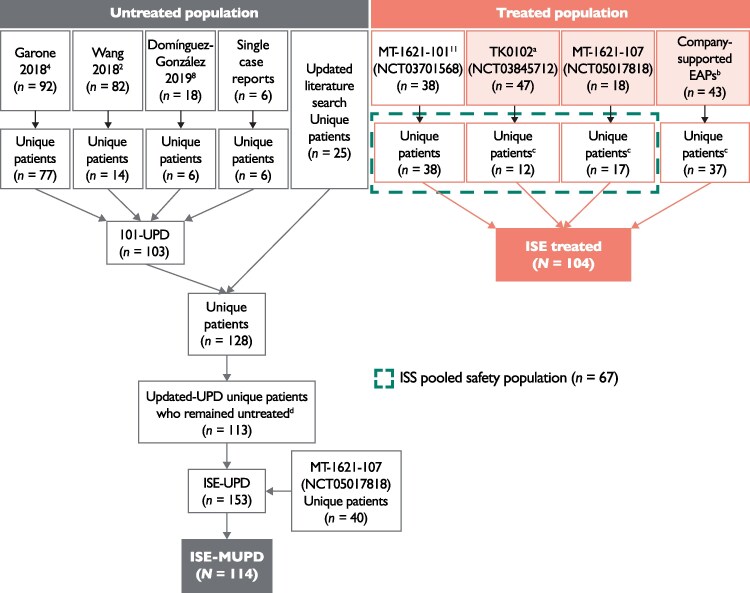
**Study analysis populations.** Individual patient data described in each publication/source were cross-referenced to remove duplicates, ensuring data were from unique patients. The untreated group (ISE-MUPD) is only used in comparative survival analyses versus the treated group (ISE treated); all other outcomes were assessed according to status before and after treatment start. Boxes with a light background indicate datasets being reported for the first time; data from some of the patients who were included in the TK0102 study were previously published.^18 a^TK0102 (data cut-off date: 15 March 2024). ^b^EAPs (data cut-off date: 1 March 2024). ^c^Patients who received treatment in another study were not included to avoid the duplication of data. ^d^Of 128 unique patients sourced from literature, 1 patient without genetic confirmation of a TK2d diagnosis was excluded from the updated-UPD group, while 14 patients were excluded as they later received treatment. EAPs, Expanded Access programs; ISE, Integrated Summary of Efficacy; ISS, Integrated Summary of Safety; UPD, Untreated Patient Database; MUPD, modified Untreated Patient Database.

Schematics for the ISE and ISS populations (henceforth referred to as the efficacy and safety populations, respectively) are presented in Fig. 1. Data were collected prospectively [TK0102 (data cut-off date: 15 March 2024); EAPs (data cut-off date: 1 March 2024)] or retrospectively (all other sources: peer-reviewed publications and clinician-reported information). Inclusion and exclusion criteria were specific to each source study (Supplementary Fig. 1).

For the treated population, data for unique patients were compiled from MT-1621-101 (data published previously),^12^ TK0102 (data from some of the patients included in this study were published previously),^18^ MT-1621-107 and EAPs (first report of these data); patients in multiple studies were only included once (Fig. 1). The main eligibility criteria of the studies constituting the dataset for treated patients were as follows: confirmed biallelic pathogenic *TK2* variants; absence of other genetic or polygenic disease; and treatment of TK2d with nucleos(t)ides [non-good manufacturing practice (GMP)-grade dC monophosphate and dT monophosphate; non-GMP dC and dT; or doxecitine and doxribtimine (GMP-grade dC and dT)]. Additional criteria for the retrospective studies were the availability of medical records or at a minimum information pertaining to survival. Written informed consent was obtained for all other patients as a prerequisite of inclusion in the clinical development programme. Approvals from respective institutional review boards and ethics committees were obtained for studies in the clinical development programme, which were conducted in accordance with the Declaration of Helsinki.

For the survival analyses, the database for the untreated population was generated by gathering information from a comprehensive literature search for case studies and reviews of patients with TK2d. This population was supplemented with data from untreated patients from a retrospective chart review study (MT-1621-107). Data were cross-referenced between sources to remove duplicate patients. To be included in the untreated population, individual-level patient data and genetic confirmation of *TK2* pathogenic variants were required. As these de-identified data were sourced from the public domain, it was not possible to seek additional informed consent for these untreated patients. Patients who received treatment at any time were excluded from the untreated arm, even if data were available for the pretreatment period, ensuring this population consisted of untreated patients only. Data from unique untreated patients comprised the ISE-Untreated Patient Database (ISE-UPD). The ISE-UPD was further modified to exclude: (i) all untreated patients who died or were censored before the earliest age at which the youngest patient commenced treatment in any study (0.69 years); (ii) untreated patients whose records did not include age of symptom onset or either age at death or age last known alive (after onset of symptoms). This generated the ISE-modified Untreated Patient Database (ISE-MUPD) for use in comparative survival analyses.

The safety analysis constitutes a subset of the treated arm (Fig. 1); no safety data were available for untreated patients. Analyses included patients with TK2d enrolled in MT-1621-101, TK0102 and MT-1621-107. Data collection in the retrospective study MT-1621-107 was limited; therefore, results of some safety analyses only included data from MT-1621-101 and TK0102.

Data are reported for subgroups defined by age of symptom onset. The main subgroups reported are patients with age of symptom onset ≤12 and >12 years. The age-of-symptom-onset-≤12-years subgroup was also subdivided according to onset ≤2 years and >2 to ≤12 years.^2-4^

### Outcomes

The primary efficacy outcome was survival, defined as time to death from symptom onset and from treatment start, and the number and proportion of deaths over the follow-up period. Secondary outcomes were the assessment of developmental motor milestones [attainment, loss or regain of key developmental motor milestones reflective of those described by the World Health Organization (ability to hold head upright, sit upright, stand assisted and unassisted, walk assisted and unassisted, climb stairs assisted and unassisted and run)],^19^ ventilatory support use (invasive or non-invasive), and enteral feeding tube use (nasogastric tube, gastrostomy tube) before and after treatment initiation. Survival outcomes were compared with those of an external untreated control group (ISE-MUPD, henceforth referred to as the untreated group). For the safety analysis, safety and tolerability of pyrimidine nucleos(t)ides were assessed.

Safety outcomes included treatment-emergent adverse events (TEAEs), defined as an adverse event absent before treatment start or already present before treatment start that worsened in severity or frequency after treatment initiation. Reference ranges for all laboratory results were based on the American Board of Internal Medicine Laboratory Test Reference Ranges, July 2021, with the Royal College of Paediatrics and Child Health reference ranges based on age for paediatric patients. Potential Hy’s Law cases, defined as patients with alanine aminotransferase (ALT) or aspartate aminotransferase (AST) values at least three times the upper limit of normal (ULN), total bilirubin at least two times ULN and alkaline phosphatase (ALP) less than two times ULN (based on the same blood draw of ALT, AST, total bilirubin and ALP while receiving study drug or up to 28 days after last received dose) were used to assess the potential for study-drug-induced liver injury. Specific liver-related laboratory outcomes were assessed and aligned with the hepatocellular injury and liver function tests and values highlighted in US FDA guidance on detecting potential drug-induced liver injury.^20^ For ECG data, an independent centralized ECG overread of all collected data was conducted to account for differences in the methodology between studies.

### Statistical analyses

Treatment efficacy was evaluated by analysing survival (comparing treated and untreated patients using matched-pairs methodology) and differences between the before and after treatment initiation periods in developmental motor milestones, ventilatory support and feeding support (treated group only). Demographic and clinical characteristics were summarized with descriptive statistics. Continuous variables were summarized using mean, standard deviation, median, range, Q1 and Q3; categorical variables were summarized as frequency or percentage. Safety data were summarized in the pooled safety population (patients with TK2d who received at least one dose of pyrimidine nucleos(t)ide therapy). The individual studies did not have a control arm.

The primary analysis was to assess survival with matched-pair data from the treated and untreated groups. Cox proportional hazard models (with and without the Firth’s correction), with and without age of symptom onset as covariate, and marginal Cox models (herein collectively called Cox models) were used to assess risk of death. Restricted mean survival time (RMST) was estimated from the area under the Kaplan–Meier curve up to a specific time point (30, 20 and 10 years after TK2d symptom onset, and 6, 4 and 2 years after treatment start). RMST analyses were used to summarize improvement in survival time with treatment.

Matched pairs for analyses were generated by selecting each treated patient and the corresponding untreated match from the same age-of-symptom-onset subgroup (≤2, >2 to ≤12, ≤12, >12 years). The matched pairs were selected based on different matching selection methods (random, 50th percentile, 75th percentile and 100th percentile), after sorting untreated patients based on survival time and treated patients based on treatment time, resulting in increasingly conservative analyses (Supplementary Fig. 2). Here, we report the results from models fitted for the 50th percentile matched-pair selection method using the Firth’s correction.

Multiple survival analyses were performed to evaluate whether estimated treatment effects were consistently obtained and therefore robust to bias and confounding factors. Analyses included a strict matching methodology, covariate adjustment and stratification, and sensitivity analyses were performed considering additional confounders such as birth order, geographic region of residence and birth year.

All statistical analyses were performed using SAS software, version 9.4 (SAS Institute, Inc., Cary, NC, USA). SAS codes and R scripts created specifically for the survival analyses can be found in the Supplementary materials.

## Results

### Patient baseline demographics and characteristics

Overall, 218 patients were included in efficacy analyses: 104 treated patients and 114 untreated patients. Baseline demographics and characteristics for treated and untreated patients are presented in Table 1 and Supplementary Table 2. The pooled safety population comprised 67 patients; some safety outcomes are reported for 50 patients when data from the 17 patients in MT-1621-107 were not available (Supplementary Table 3).

**Table 1:** Baseline demographics and characteristics

	Overall		Patients with age of TK2d symptom onset ≤12 years		Patients with age of TK2d symptom onset >12 years	
	Treated	Untreated	Treated	Untreated	Treated	Untreated
*N*	104	114	82	93	22	21
Sex, *n* (%)						
Male	55 (52.9)	55 (48.2)	46 (56.1)	49 (52.7)	9 (40.9)	6 (28.6)
Female	49 (47.1)	59 (51.8)	36 (43.9)	44 (47.3)	13 (59.1)	15 (71.4)
Race,^a^ *n* (%)						
White	87 (83.7)	34 (29.8)	67 (81.7)	24 (25.8)	20 (90.9)	10 (47.6)
Other	13 (12.5)	2 (1.8)	11 (13.4)	2 (2.2)	2 (9.1)	0 (0)
Not reported	4 (3.8)	78 (68.4)	4 (4.9)	67 (72.0)	0 (0)	11 (52.4)
Ethnicity, *n* (%)						
Hispanic or Latino	30 (28.8)	13 (11.4)	30 (36.6)	12 (12.9)	0 (0)	1 (4.8)
Not Hispanic or Latino	61 (58.7)	24 (21.1)	41 (50.0)	14 (15.1)	20 (90.9)	10 (47.6)
Not reported	13 (12.5)	77 (67.5)	11 (13.4)	67 (72.0)	2 (9.1)	10 (47.6)
Geographic region of residence,^a^ *n* (%)						
Europe	43 (41.3)	32 (28.1)	27 (32.9)	20 (21.5)	16 (72.7)	12 (57.1)
Rest of the world	61 (58.7)	51 (44.7)	55 (67.1)	47 (50.5)	6 (27.3)	4 (19.0)
Not reported	0 (0)	31 (27.2)	0 (0)	26 (28.0)	0 (0)	5 (23.8)
Age of TK2d symptom onset, years						
Median	1.96	1.52	1.50	1.33	27.06	40.00
Min, max	0.01, 60.30	0.00, 72.00	0.01, 11.67	0.00, 11.00	12.36, 60.30	12.04, 72.00
Q1, Q3	1.17, 5.69	0.92, 7.00	1.08, 2.41	0.75, 2.49	17.79, 39.98	23.49, 40.41
Age at first treatment (any treatment), years						
Median	7.34	NA	4.26	NA	50.86	NA
Min, max	0.69, 74.01	NA	0.69, 35.52	NA	17.30, 74.01	NA
Q1, Q3	2.58, 26.28	NA	2.11, 10.49	NA	31.78, 58.66	NA

min, minimum; max, maximum; NA, not applicable; Q1, quartile 1; Q3, quartile 3; TK2d, thymidine kinase 2 deficiency.

^a^Owing to the ultra-rare nature of TK2d and the small number of patients, some details relating to race and country of residence were grouped for reporting purposes to minimize risk of patient identification.

For treated patients, median (Q1, Q3) age of symptom onset and age at first treatment were 1.96 (1.17, 5.69) years and 7.34 (2.58, 26.28) years, respectively; age of symptom onset was similar for untreated patients [1.52 (0.92, 7.00) years] (Table 1). Most patients had an age of symptom onset ≤12 years in both the treated [82/104 (78.8%)] and untreated [93/114 (81.6%)] populations. Of those with age of symptom onset ≤12 years, most had an age of symptom onset ≤2 years [treated, 56/82 (68.3%); untreated, 69/93 (74.2%)] (Supplementary Table 2).

Baseline demographics and characteristics of the age-of-symptom-onset-≤12-years subgroup mirrored that of the overall population, although with earlier age of symptom onset [treated, 1.50 (1.08, 2.41) years; untreated, 1.33 (0.75, 2.49) years] and age at first treatment [treated, 4.26 (2.11, 10.49) years], as expected.

As the population was driven by those with earlier-onset disease, data are presented separately for age of symptom onset ≤12 years and >12 years; subdivisions of the ≤12 years subgroup (i.e. age of symptom onset ≤2 years and >2 to ≤12 years) are included as supplementary data.

### Summary of study drug administration in efficacy and safety populations

In the treated population, median (Q1, Q3) treatment duration was 54.76 (15.23, 78.45) months and 27.16 (3.84, 78.02) months for those with age of symptom onset ≤12 and >12 years, respectively (Table 2). In the pooled safety population, median treatment duration was 70.90 (50.65, 87.34) months, with most patients receiving treatment for 2 to <5 years [13/67 (19.4%)] or longer [40/67 (59.7%)].

**Table 2:** Summary of study drug administration

	Treated	
	Patients with age of TK2d symptom onset ≤12 years (*N* = 82)	Patients with age of TK2d symptom onset >12 years (*N* = 22)
Treatment duration, months		
Mean (SD)	51.50 (41.64)	41.17 (40.54)
Median (min, max)	54.76 (0.04, 157.07)	27.16 (0.82, 113.25)
Q1, Q3	15.23, 78.45	3.84, 78.02
Treatment duration, *n* (%)		
At least one dose	82 (100)	22 (100)
2 days to <1 month	1 (1.2)	1 (4.5)
1 to <3 month(s)	3 (3.7)	3 (13.6)
3 to <6 months	7 (8.5)	3 (13.6)
6 to <12 months	7 (8.5)	3 (13.6)
12 to <15 months	1 (1.2)	0 (0)
15 to <18 months	6 (7.3)	0 (0)
18 months to <2 years	7 (8.5)	1 (4.5)
2 to <5 years	16 (19.5)	3 (13.6)
≥5 years	33 (40.2)	8 (36.4)
Time from TK2d symptom onset to start of treatment, *n* (%)		
≤6 months	11 (13.4)	0 (0)
>6 months to ≤1 year	13 (15.9)	0 (0)
>1 to ≤5 years	29 (35.4)	2 (9.1)
>5 to ≤10 years	13 (15.9)	3 (13.6)
>10 years	16 (19.5)	17 (77.3)

min, minimum; max, maximum; Q1, quartile 1; Q3, quartile 3; SD, standard deviation; TK2d, thymidine kinase 2 deficiency.

Treatment delay occurred in both main age subgroups, with the majority of those with age of symptom onset ≤12 years and all with age of symptom onset >12 years experiencing symptoms for more than 1 year before initiating treatment (Table 2). Treatment delay was longer in the age-of-symptom-onset->12-years subgroup than in the age-of-symptom-onset-≤12-years subgroup (proportion reporting treatment delay of >10 years: 77.3 versus 19.5%).

Within the age-of-symptom-onset-≤12-years subgroup, patients with age of symptom onset ≤2 years had a shorter median treatment duration and a smaller proportion of patients reporting delays of >10 years to treatment initiation than those with age of symptom onset >2 to ≤12 years (Supplementary Table 4).

### Survival

Patient survival results are summarized in Table 3 and Supplementary Table 5. Among patients with age of symptom onset ≤12 years, the proportion of deaths was substantially lower in treated patients [3/82 patients (3.7%)] than in untreated patients [53/93 patients (57.0%)]. Fewer patients died overall in the age-of-symptom-onset->12-years subgroup; however, there were proportionally fewer deaths in treated patients [4/22 patients (18.2%)] than in untreated patients [5/21 patients (23.8%)].

**Table 3:** Summary of patient survival

	Patients with age of TK2d symptom onset ≤12 years		Patients with age of TK2d symptom onset >12 years	
	Treated	Untreated	Treated	Untreated
*N*	82	93	22	21
Reported death, *n* (%)	3 (3.7)	53 (57.0)	4 (18.2)	5 (23.8)
Age at death, years				
Mean (SD)	11.27 (17.75)	4.55 (6.27)	55.17 (13.85)	59.40 (12.03)
Median (min, max)	1.11 (0.94, 31.77)	2.64 (0.83, 33.50)	57.35 (37.20, 68.77)	64.00 (40.00, 70.00)
Q1, Q3	0.94, 31.77	1.58, 4.00	44.58, 65.76	56.00, 67.00
No reported death, *n* (%)	79 (96.3)	40 (43.0)	18 (81.8)	16 (76.2)
Age last known alive, years	*n* = 79	*n* = 40	*n* = 18	*n* = 16
Mean (SD)	12.48 (9.38)	15.10 (12.82)	49.05 (17.10)	46.97 (12.52)
Median (min, max)	9.86 (1.1, 41.0)	11.35 (0.8, 53.0)	52.08 (17.4, 80.0)	47.19 (24.0, 74.0)
Q1, Q3	5.67, 15.05	5.15, 20.46	37.19, 64.41	36.91, 54.33

min, minimum; max, maximum; Q1, quartile 1; Q3, quartile 3; SD, standard deviation; TK2d, thymidine kinase 2 deficiency.

**Figure 2:**
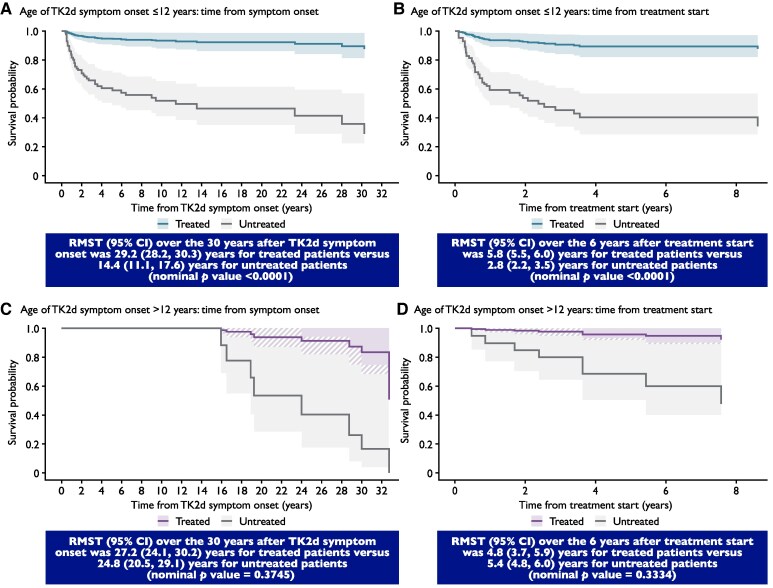
**Direct adjustment survival curves and RMSTs (from TK2d symptom onset and from treatment start; 50th-percentile matching estimated from marginal Cox models).** Direct adjustment survival curves were estimated using a Cox marginal model with age of TK2d symptom onset as strata variable. RMSTs were estimated from the area under the Kaplan–Meier curves; nominal *P*-values are not multiplicity adjusted. (**A**) Direct adjustment survival curves from symptom onset to death in treated patients (*n* censored patients = 75) versus untreated patients (*n* censored patients = 38) with age of TK2d symptom onset ≤12 years (Wald test *P* < 0.0001) and RMST over 30 years after symptom onset. (**B**) Direct adjustment survival curves from treatment start to death in treated patients (*n* censored patients = 75) versus untreated patients (*n* censored patients = 38) with age of TK2d symptom onset ≤12 years (Wald test *P* < 0.0001) and RMST over 6 years after treatment start. (**C**) Direct adjustment survival curves from symptom onset to death in treated patients (*n* censored patients = 13) versus untreated patients (*n* censored patients = 13) with age of TK2d symptom onset >12 years (Wald test *P* = 0.3305) and RMST over 30 years after symptom onset. (**D**) Direct adjustment survival curves from treatment start to death in treated patients (*n* censored patients = 13) versus untreated patients (*n* censored patients = 13) with age of TK2d symptom onset >12 years (Wald test *P* = 0.2661) and RMST over 6 years after treatment start. RMST, restricted mean survival time; TK2d, thymidine kinase 2 deficiency. Purple dash area indicates overlap between treated and untreated populations.

In the age-of-symptom-onset-≤12-years subgroup, risk of death was reduced with treatment by 92–94% (hazard ratio = 0.06–0.08) in the time from symptom onset and by 87–95% (hazard ratio = 0.05–0.13) in the time from treatment initiation (Supplementary Fig. 3). In addition, RMST (95% confidence interval) in treated patients was 29.2 (28.2, 30.3) years over the 30 years after symptom onset compared with 14.4 (11.1, 17.6) years in untreated patients (Fig. 2A; Supplementary Fig. 4A), and 5.8 (5.5, 6.0) years over the 6 years after treatment initiation compared with 2.8 (2.2, 3.5) years in untreated patients (Fig. 2B; Supplementary Fig. 4B).

In the age-of-symptom-onset->12-years subgroup, Cox models showed no difference in risk of death between treated and untreated patients (Supplementary Fig. 3). There was also no significant difference in RMST estimates between treated and untreated patients in this subgroup (Fig. 2C and D; Supplementary Fig. 4).

The same trends were observed across different matching selection methods (Supplementary Fig. 4). Sensitivity analyses were conducted to assess the impact of sex, geographic region, birth year and age of symptom onset, excluding treated and untreated patients who required partial date imputation for any survival analysis key dates. The subgroup analyses carried out were not large enough to be informative; therefore, these are not detailed here.

Within the age-of-symptom-onset-≤12-years subgroup, the proportion of patients who died was lower with treatment than without in both the age-of-symptom-onset-≤2-years and >2 to ≤12 years subgroups. The benefit of treatment was more pronounced in the age-of-symptom-onset-≤2-years subgroup than in the >2 to ≤12 years subgroup in terms of overall numbers, Cox models and RMST estimates, with the low number of patients and events confounding the analysis of the age-of-symptom-onset->2-to-≤12-years subgroup (Supplementary Table 5; Supplementary Figs. 3 and 4).

### Developmental motor milestones

**Figure 3:**
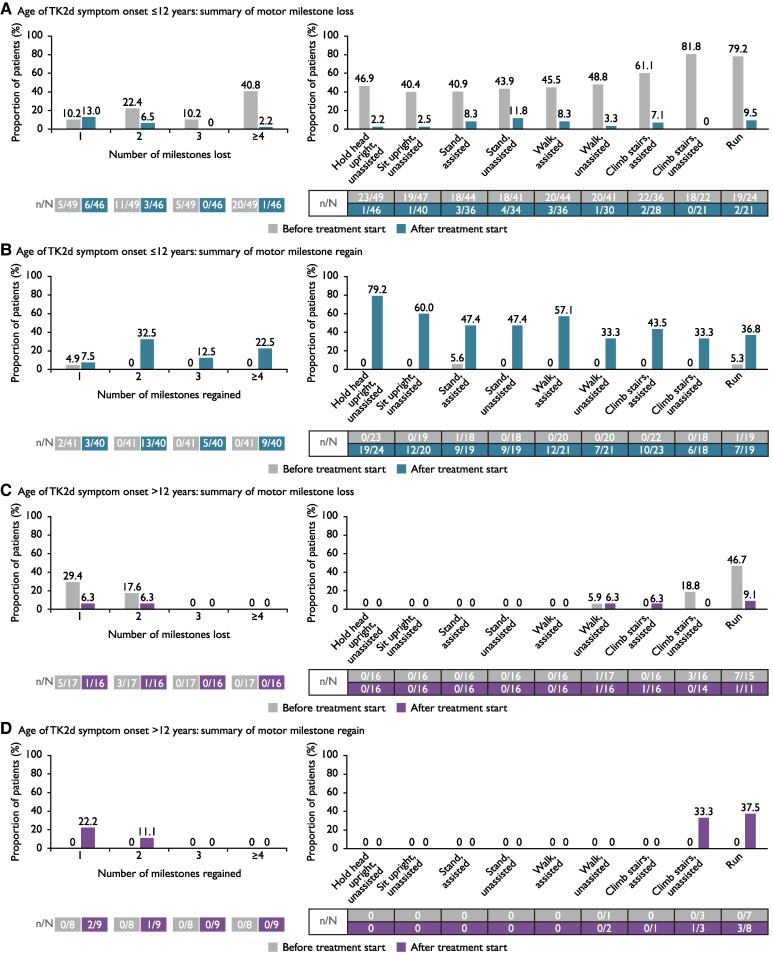
**Developmental motor milestone loss and regain before and after treatment initiation.** (**A**) Proportion of patients with age of symptom onset ≤12 years who lost developmental motor milestones before and after treatment start. (**B**) Proportion of patients with age of symptom onset ≤12 years who regained previously lost developmental motor milestones before and after treatment start. (**C**) Proportion of patients with age of symptom onset >12 years who lost developmental motor milestones before and after treatment start. (**D**) Proportion of patients with age of symptom onset >12 years who regained previously lost developmental motor milestones before and after treatment start. TK2d, thymidine kinase 2 deficiency.

The evolution of developmental motor milestones in treated patients before and after treatment initiation is summarized in Fig. 3. In the age-of-symptom-onset-≤12-years subgroup, of the patients who initially achieved at least one motor milestone, 41/49 (83.7%) lost at least one motor milestone, and 20/49 (40.8%) lost at least four motor milestones, before treatment initiation. The most common motor milestone lost before treatment initiation was ability to climb stairs unassisted [18/22 (81.8%)], followed by ability to run [19/24 (79.2%)]. After treatment initiation, motor milestone loss was less frequent: 10/46 patients (21.7%) lost at least one motor milestone and 1/46 patients (2.2%) lost at least four.

Within the age-of-symptom-onset-≤12-years subgroup, the proportion of patients who lost milestones was notable before treatment initiation in both the age-of-symptom-onset-≤2-years and >2 to ≤12 years subgroups. After treatment initiation, milestone loss was markedly reduced in both subgroups (Supplementary Fig. 5).

In the age-of-symptom-onset-≤12-years subgroup, only 2 (4.9%) of the 41 patients who had lost motor milestones spontaneously regained one lost motor milestone before treatment (Fig. 3B). After treatment initiation, most patients regained at least one motor milestone [30/40 (75.0%)] and 9/40 (22.5%) regained at least four; motor milestones were regained across the range of those assessed. A similar trend occurred in both the age-of-symptom-onset-≤2-years and >2 to ≤12 years subgroups, with motor milestone regain rarely reported before treatment but frequently reported across the range of those assessed after treatment initiation (Supplementary Fig. 5).

In the age-of-symptom-onset->12-years subgroup, the loss of motor milestones before treatment initiation was less frequent than in the age-of-symptom-onset-≤12-years subgroup, with 8/17 patients (47.1%) losing at least one motor milestone and no patients losing at least four motor milestones. Within this subgroup, the most common motor milestone lost was ability to run [7/15 (46.7%)]. After treatment initiation, only 2/16 patients (12.5%) lost at least one motor milestone (Fig. 3C).

Before treatment, no patients in the age-of-symptom-onset->12-years subgroup spontaneously regained motor milestones. After treatment initiation, 3/9 patients (33.3%) in this subgroup regained at least one motor milestone, namely, ability to climb stairs unassisted [1/3 (33.3%)] and/or to run [3/8 (37.5%)] (Fig. 3D).

### Ventilatory and feeding tube support

Data for ventilatory support were not available for 29/82 patients with age of symptom onset ≤12 years (35.4%) and for 5/22 patients with age of symptom onset >12 years (22.7%; Table 4; Supplementary Table 6). Before treatment, 31/82 treated patients in the age-of-symptom-onset-≤12-years subgroup (37.8%) and 9/22 in the age-of-symptom-onset->12-years subgroup (40.9%) used ventilatory support. In the age-of-symptom-onset-≤12-years subgroup, non-invasive biphasic positive airway pressure or continuous airway pressure was the most common mode of ventilatory support [20/31 (64.5%)] at treatment start. Of those who did not use ventilatory support at treatment start, 4/22 (18.2%) in the age-of-symptom-onset-≤12-years subgroup and 3/8 (37.5%) in the age-of-symptom-onset->12-years subgroup started ventilatory support after treatment initiation. Although no patients in the age-of-symptom-onset-≤12-years subgroup discontinued ventilatory support before treatment initiation, 6/35 patients (17.1%) using support at any time after treatment initiation later discontinued support. In the age-of-symptom-onset->12-years subgroup, no patients discontinued ventilatory support before treatment initiation and 1/12 (8.3%) discontinued ventilatory support after treatment initiation. Among patients who initiated ventilatory support before treatment initiation, 5/31 (16.1%) and 1/9 (11.1%) decreased number of hours of ventilatory support after treatment initiation in the age-of-symptom-onset-≤12-years and >12 years subgroups, respectively.

**Table 4:** Summary of ventilatory and enteral feeding tube support use in treated patients in the age-of-symptom-onset-≤12-years and age-of-symptom-onset->12-years subgroups before and after treatment

	Patients with age of TK2d symptom onset ≤12 years (*N* = 82)		Patients with age of TK2d symptom onset >12 years (*N* = 22)	
	Before treatment	After treatment	Before treatment	After treatment
Summary of ventilatory support				
Initiated ventilatory support, *n/N* (%)	31/82 (37.8)	4/22^a^ (18.2)	9/22 (40.9)	3/8^a^ (37.5)
Discontinued ventilatory support, *n/N* (%)	0/31^b^ (0)	6/35^c^ (17.1)	0/9^b^ (0)	1/12^c^ (8.3)
Hours of ventilatory support per day (last observation)				
*n*	28	17	9	6
Median (min, max)	11.0 (8.0, 24.0)	8.0 (0, 24.0)	9.0 (7.0, 24.0)	8.0 (0.0, 13.0)
Q1, Q3	8.0, 24.0	0.0, 14.0	8.0, 10.0	7.0, 11.0
No ventilatory support data collected, *n* (%)	29 (35.4)		5 (22.7)	
Summary of enteral feeding tube support				
Enteral feeding tube inserted, *n/N* (%)	20/82 (24.4)	4/33^a^ (12.1)	4/22 (18.2)	1/12^a^ (8.3)
Reason for tube insertion,^d^ *n/N* (%)				
Supplemental oral intake	8/20 (40.0)	3/4 (75.0)	1/4 (25.0)	0 (0)
Dysphagia	17/20 (85.0)	1/4 (25.0)	4/4 (100)	1/1 (100)
Other	3/20 (15.0)	1/4 (25.0)	0 (0)	0 (0)
Enteral feeding tube removed, *n/N* (%)	1/20^b^ (5.0)	4/23^c^ (17.4)	0/4^b^ (0)	0/5^c^ (0)
Reason for tube removal,^d^ *n/N* (%)				
Improvement not otherwise specified	0/1 (0)	1/4 (25.0)	0 (0)	0 (0)
Improved dysphagia and oral feeding	0/1 (0)	1/4 (25.0)		
Improved dysphagia	0/1 (0)	1/4 (25.0)	0 (0)	0 (0)
Improved oral feeding	1/1 (100)	0/4 (0)	0 (0)	0 (0)
Other	1/1 (100)	0/4 (0)	0 (0)	0 (0)
Adverse device event	0/1 (0)	1/4 (25.0)	0 (0)	0 (0)
No enteral feeding tube support data collected, *n* (%)	30 (36.6)		6 (27.3)	

min, minimum; max, maximum; Q1, quartile 1; Q3, quartile 3; TK2d, thymidine kinase 2 deficiency.

^a^ *N* is patients with available data not using support before treatment initiation who were at risk of starting support after treatment initiation.

^b^ *N* is patients using support before treatment initiation who were at risk of discontinuing support.

^c^ *N* is patients using support at any time after treatment initiation who were at risk of discontinuing support.

^d^Patients could be counted in more than one category for reasons for tube insertion and tube removal.

Data for feeding tube support were not available for 30/82 patients with age of symptom onset ≤12 years (36.6%) and for 6/22 patients with age of symptom onset >12 years (27.3%). In total, 20/82 patients (24.4%) and 4/22 patients (18.2%) had an enteral feeding tube before treatment initiation in the age-of-symptom-onset-≤12-years and >12 years subgroups, respectively (Table 4). The reasons for enteral feeding tube insertion before and after treatment are included in Table 4. In the age-of-symptom-onset-≤12-years subgroup, feeding support was discontinued before treatment initiation by one patient [1/20 (5.0%)] and after treatment initiation by four patients [2/19 patients who had support at treatment start (10.5%) and 2/4 patients who initiated support during treatment (50.0%)].

Within the age-of-symptom-onset-≤12-years subgroup, similar results were found in both the age-of-symptom-onset-≤2-years and >2-to-≤12-years subgroups, with limited changes observed in ventilatory or feeding support (Supplementary Table 6).

### Safety

The pooled safety population included 67 patients with TK2d. Some safety variables were not available in MT-1621-107; therefore, some safety outcomes are reported for 50 patients when data from the 17 patients in MT-1621-107 were not available.

The majority of patients in the pooled safety population [40/67 (59.7%)] had a treatment exposure of at least 5 years, with an overall cumulative exposure of 345.0 patient-years.

In the pooled safety population, all 50 patients for whom TEAE data were available reported at least one TEAE and 43/50 (86.0%) had at least one drug-related TEAE per Investigator assessment (Table 5; Supplementary Table 7). Most TEAEs did not lead to discontinuation or dose reduction; 9/67 patients (13.4%) discontinued treatment as a result of a TEAE and 16/67 (23.9%) reported at least one TEAE that led to dose reduction (Supplementary Table 8). The most frequently reported TEAE was diarrhoea [43/50 (86.0%)], which was generally mild or moderate (Supplementary Table 9) and resolved with dose reduction [14/67 patients (20.9%) had a dose reduction]. Diarrhoea was considered study-drug-related per the investigator in 40/50 patients (80.0%); one event of diarrhoea was considered serious in one patient [1/50 (2.0%)], who continued treatment without further reported events. Non-serious events of diarrhoea led to treatment discontinuation in 2/67 patients (3.0%). Adverse drug reactions identified as potential side effects of doxecitine and doxribtimine treatment were diarrhoea [43/50 (86.0%)], vomiting [14/50 (28.0%)], ALT increased [14/50 (28.0%)], AST increased [11/50 (22.0%)] and abdominal pain [including abdominal pain upper; 13/50 (26.0%)]. At baseline (which may include earliest measure after treatment start when measure before treatment was not available), there were 27/50 patients (54.0%) with elevated ALT (ALT >1–3 times ULN, *n* = 18; ALT of 3–5 times ULN, *n* = 6; ALT of 5–20 times ULN, *n* = 3) and 21/50 patients (42.0%) with elevated AST (AST greater than one to three times ULN, *n* = 15; AST of three to five times ULN, *n* = 7). Among patients with at least 4 weeks of treatment exposure, 9/50 (18.0%) experienced ALT or AST increases greater than three times ULN; however, no events were considered as potential drug-induced liver injury and no patients met the laboratory criteria for potential Hy’s Law. Most events of ALT or AST increase resolved without dose change: 13/14 ALT elevation events (92.9%) and 9/11 AST elevation events (81.8%). Based on the Common Terminology Criteria for Adverse Events, the severity of TEAEs was reported as follows: mild (Grade 1) in 5/50 patients (10.0%); moderate (Grade 2) in 16/50 patients (32.0%); severe (Grade 3) in 21/50 patients (42.0%), life-threatening (Grade 4) in 7/50 patients (14.0%) and fatal (Grade 5) in 1/50 patients (2.0%) (Supplementary Table 9).

**Table 5:** Summary of TEAEs in at least 20% of patients in the pooled safety population

	MT-1621-101 and TK0102 (*N* = 50)	MT-1621-101, TK0102 and MT-1621-107 (*N* = 67)
Patients with at least one TEAE, *n* (%)	50 (100)	—
Patients with TEAE related to study drug, *n* (%)	43 (86.0)	—
Patients with TEAE leading to study drug discontinuation, *n* (%)	3 (6.0)	9 (13.4)
Patients with TEAE leading to dose reduction, *n* (%)	14 (28.0)	16 (23.9)
Patients with TEAEs by system organ class and preferred term, *n* (%)		
Gastrointestinal disorders	46 (92.0)	—
Diarrhoea	43 (86.0)	—
Vomiting	14 (28.0)	—
Abdominal pain	10 (20.0)	—
General disorders and administration-site conditions	29 (58.0)	—
Pyrexia	20 (40.0)	—
Infections and infestations	43 (86.0)	—
Upper respiratory tract infection	19 (38.0)	—
COVID-19	18 (36.0)	—
Pneumonia	10 (20.0)	—
Respiratory tract infection	10 (20.0)	—
Injury, poisoning and procedural complications	20 (40.0)	—
Investigations	35 (70.0)	—
ALT increased	14 (28.0)	—
AST increased	11 (22.0)	—
Blood creatine phosphokinase increased	10 (20.0)	—
Musculoskeletal and connective tissue disorders	22 (44.0)	—
Nervous system disorders	22 (44.0)	—
Headache	13 (26.0)	—
Respiratory, thoracic and mediastinal disorders	34 (68.0)	—
Rhinorrhoea	15 (30.0)	—
Cough	11 (22.0)	—
Skin and subcutaneous tissue disorders	17 (34.0)	—

All adverse event terms were coded to Medical Dictionary for Regulatory Activities version 26.0.

ALT, alanine aminotransferase; AST, aspartate aminotransferase; TEAE, treatment-emergent adverse event.

Treatment-emergent serious adverse events (TESAEs) were experienced by 28/50 patients (56.0%), the most frequently reported being pneumonia [7/50 (14.0%)], acute respiratory failure [5/50 (10.0%)], femur fracture [4/50 (8.0%)], dysphagia [3/50 (6.0%)] and pneumonia aspiration [3/50 (6.0%)]. Few patients experienced TESAEs that were study-drug-related [4/50 (8.0%)]. In total, 4/67 patients (6.0%) discontinued treatment owing to TESAEs. In total, 3/67 patients (4.5%) experienced a fatal TESAE. The events leading to death were seizures, disease progression and unknown causes, which were not considered related to the study drug.

An assessment of paediatric growth, neurodevelopment, behaviour and endocrine (PGNBE)-related adverse events in patients aged ≤2 years at first treatment did not indicate a specific risk of doxecitine and doxribtimine for PGNBE. However, the low number of patients in this subgroup (*n* = 9) may limit generalization of these results.

No clinically meaningful changes in vital signs were observed over time. Minor and transient fluctuations were observed in mean systolic and diastolic blood pressure, heart rate, respiratory rate and temperature. A review of ECG data in MT-1621-101 and TK0102 demonstrated no evidence of clinically significant effects of doxecitine and doxribtimine on QT interval corrected for heart rate or other parameters.

## Discussion

TK2d is a severe, devastatingly progressive and often fatal disease affecting almost every aspect of patients’ lives.^21^ These analyses combined multiple data sources to assess efficacy and safety of pyrimidine nucleos(t)ide treatment in a large, integrated population of patients with TK2d. Our findings provide a clear indication that treatment improves both survival and functional outcomes in patients with TK2d and age of symptom onset ≤12 years, with some indication of functional benefits also apparent in those with age of symptom onset >12 years. Doxecitine and doxribtimine is the first FDA- and EMA-approved treatment for TK2d, and it targets the underlying disease pathology.

A key finding of our pooled analysis is that pyrimidine nucleos(t)ide treatment decreased mortality in patients with TK2d. Symptoms of TK2d tend to progress more rapidly in individuals who experience disease onset at a younger age,^3,4,8^ and the greatest survival benefit of pyrimidine nucleos(t)ide treatment in our analysis was in the age-of-symptom-onset-≤12-years subgroup. These patients had significantly reduced risk of death and longer time to death from symptom onset when compared with untreated patients. It should be noted that this benefit was driven by the age-of-symptom-onset-≤2-years subgroup, which may partly reflect the higher frequency of deaths among untreated patients and larger number of patients overall in this subgroup compared with the age-of-symptom-onset->2-to-≤12-years subgroup. Although there was no statistically significant difference in risk of death between treated and untreated patients with age of symptom onset >12 years, a lower proportion of patients died and the time to death from symptom onset was longer in treated patients than in untreated patients. However, analyses in this subgroup may have been underpowered given the low numbers of patients and matching pairs available for analysis, as well as the lower mortality. In addition, a longer observation period is required to record deaths associated with a slower disease progression, and the 6-year observation period for analysing RMST from treatment start may therefore have been insufficient to detect a treatment benefit in this subgroup.

The consequences of TK2d can be devastating for those with the disease and their caregivers. People living with TK2d report significantly worse quality-of-life scores than those of the general population on global physical and mental health, physical function, pain interference, fatigue, anxiety and social domains.^21^ Patients with TK2d and early symptom onset (i.e. ≤1 or ≤12 years) typically do not achieve normal developmental motor milestones, or they lose those already gained, and patients with late symptom onset (i.e. >12 years) lose functional independence as they develop problems breathing and eating.^4,11^ A study on the disease course of untreated patients with TK2d showed that most patients with age of symptom onset ≤12 years lost at least one previously acquired motor milestone.^11^ Our findings in the age-of-symptom-onset-≤12-years subgroup (and both subgroups therein) demonstrate that pyrimidine nucleos(t)ide treatment can address this key component of TK2d pathophysiology. Although definitively attributing lack of motor milestone achievement to TK2d in the youngest patients may be challenging, the loss of a motor milestone is never a normal occurrence in healthy individuals, and in TK2d, spontaneous regain of a lost motor milestone is rare.^11^ The reduced frequency of motor milestone loss and corresponding regain across the full range of motor milestones after treatment initiation with pyrimidine nucleos(t)ide is striking and suggests that treatment substantially slows or stabilizes disease progression and aids significant regain of function. Although the impact of treatment on motor function was not as marked in the age-of-symptom-onset->12-years subgroup, it should be noted that these patients lost the ability to run, climb stairs unassisted and walk unassisted before treatment initiation, and those same milestones were in some cases regained after treatment initiation. Additionally, patients in the age-of-symptom-onset->12-years subgroup can have clinically significant muscle weakness while retaining the specific motor functions explored here. The specific motor milestones assessed in the present analysis did not measure muscle strength and thus may not reflect the severity of motor impairment.

The disease burden in people with TK2d is not only exemplified by the loss of motor milestones but also by the use of ventilatory and feeding support, reflecting the progressive respiratory and oropharyngeal muscle weakness that people with TK2d experience.^4^ The use of ventilatory and feeding support has been reported across all age-of-symptom-onset subgroups.^11^ The results presented here suggest a stabilization of these aspects in treated patients, with a reduction in the level of support required in some patients receiving pyrimidine nucleos(t)ides. Considering the relentlessly progressive nature of TK2d,^3,11^ the stabilization or limited progression seen in this analysis is clinically important and suggests that doxecitine and doxribtimine treatment may provide additional benefits to people with TK2d beyond the positive impact on survival. However, the use of ventilatory and feeding support may vary across different geographic regions and healthcare systems, therefore, changes in the level of use should be considered an indirect measure of potential improvements.

In the safety analysis, pyrimidine nucleos(t)ide treatment was generally well tolerated by patients with TK2d and had an acceptable safety profile consistent with previous reports.^3,12,22^ All treated patients had at least one TEAE, but the majority did not lead to discontinuation of treatment. Diarrhoea was the most common TEAE [43/50 patients (86.0%)], and most events were considered to be related to treatment; only one case was considered serious. Most patients who experienced transaminase elevations following treatment with pyrimidine nucleos(t)ides had increases that were lower than three times the ULN. Although 13 patients had elevations of transaminases greater than three times the ULN, definitive causality could not be established, because of concomitant medications and potential disease-related effects. Mitochondrial diseases, including TK2d, are known to be associated with elevations in transaminases,^2,3^ potentially linked to a mitochondria-related effect on the liver and/or disease-related muscle injury. Patients with TK2d may therefore have pre-existing transaminase elevations to varying degrees due to disease-related muscle injury. The low rate of patients experiencing drug-related TESAEs, when compared with the overall number of reported TESAEs, supports the perspective that the high rate of TESAEs in patients with TK2d may reflect other factors including the natural history of the disease. Of the 67 patients in the pooled safety population, three patients died; none of these deaths were considered related to treatment, and in two cases, the patients had been treated for only up to one month before death, limiting the opportunity to benefit from treatment.

The results of this pooled analysis suggest a positive effect of pyrimidine nucleos(t)ide therapy on survival and functional outcomes; however, some limitations should be considered when interpreting these results. A traditional randomized controlled trial to assess the efficacy and safety of pyrimidine nucleos(t)ide therapy was not possible owing to the rarity of the disease and the relatively long trial duration required to show a survival benefit, especially for those with age of symptom onset >12 years. Additionally, given initial reports of treatment benefit in compassionate use for people with TK2d and severe disease,^22^ a classical clinical trial may not be considered ethical. The use of pooled data from a mixture of heterogeneous sources brings an inherent risk of bias. It should also be noted that not all data were available for each patient because of the retrospective nature of some of the data sources, the variable nature of the disease, variation in the functional abilities of patients and clinical site preference or experience in conducting specific assessments. To address these challenges, robust objective clinical outcomes were selected to assess potential treatment effects, and the matched-pair strategy with multiple matching selection methods for assessing survival gives additional confidence in the primary survival outcomes. In addition, considering the ultra-rare nature of TK2d, integrating data from up to 218 patients currently represents most of the people with TK2d identified globally. As such, the sample size alone lends weight to the descriptive data presented here. For the subgroup with age of symptom onset >12 years, prospective data involving a larger number of patients is needed to better establish treatment benefits. The current data show a tendency towards stabilization in treated patients many years after symptom onset, but it is possible that people with TK2d treated earlier may experience clearer improvements. In addition, in this subgroup of patients with slowly progressive myopathy, detailed studies on motor function, fatigue and respiratory function may more accurately measure clinical improvements after treatment, rather than relying solely on major changes like regain of motor milestones.

## Conclusion

We provide evidence that pyrimidine nucleos(t)ide treatment reduces risk of death, increases survival time, reduces the risk of losing motor milestones and can result in the regain of lost motor milestones in people with TK2d, especially in those with age of symptom onset ≤12 years (a plain language summary of these findings is provided in the Supplementary materials to support understanding among patients, families and non-specialist audiences). Bearing in mind the major burden TK2d places on patients, caregivers and the community, in relation to both quality of life and healthcare resource utilization costs,^23^ our findings have important implications for addressing the critical unmet need of those living with TK2d.

## Supplementary Material

fcag201_Supplementary_Data

## Data Availability

Data from non-interventional studies are outside of UCB’s data sharing policy and are unavailable for sharing. The data are not publicly available as they contain information that could compromise the privacy of participants.
