# Supplementary material for: Efficacy and safety of pyrimidine nucleos(t)ide therapy in thymidine kinase 2 deficiency
Source: Brain Commun. 2026 Jun 3;8(3):fcag201. doi: 10.1093/braincomms/fcag201 (PMC13273412; doi:10.1093/braincomms/fcag201)
Supplement: fcag201_Supplementary_Data [file fcag201_supplementary_data.docx]

# Supplementary material

**Figures and Tables**

**Supplementary Figure 1** **Designs of the source studies for the efficacy and safety pooled analysis**


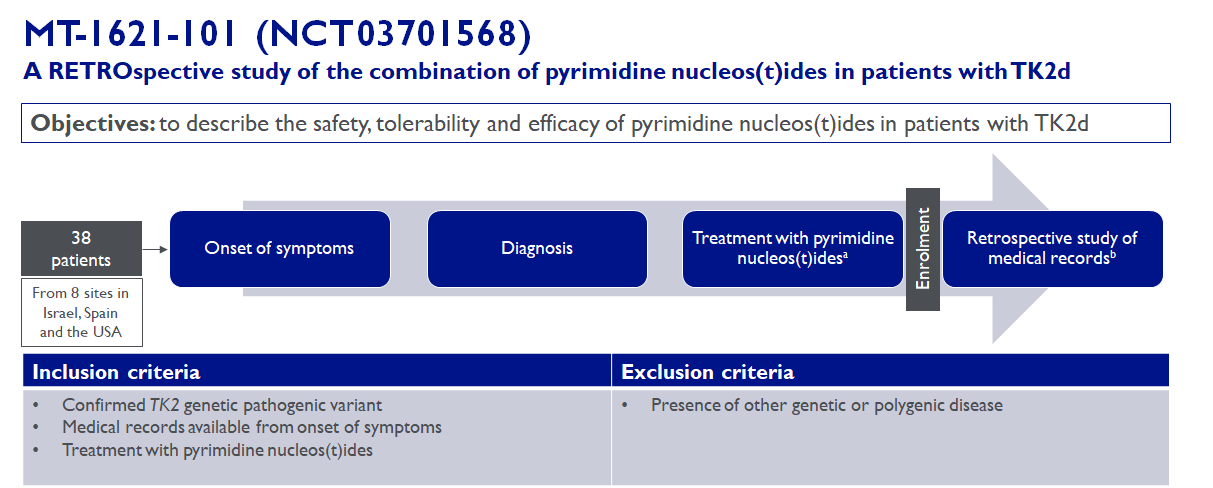


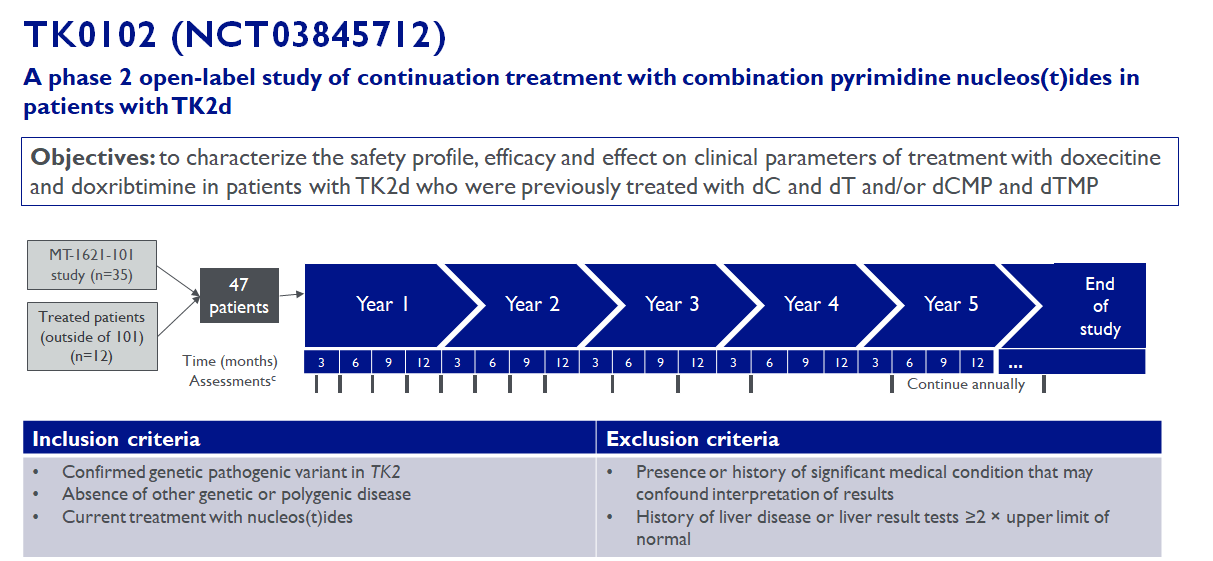


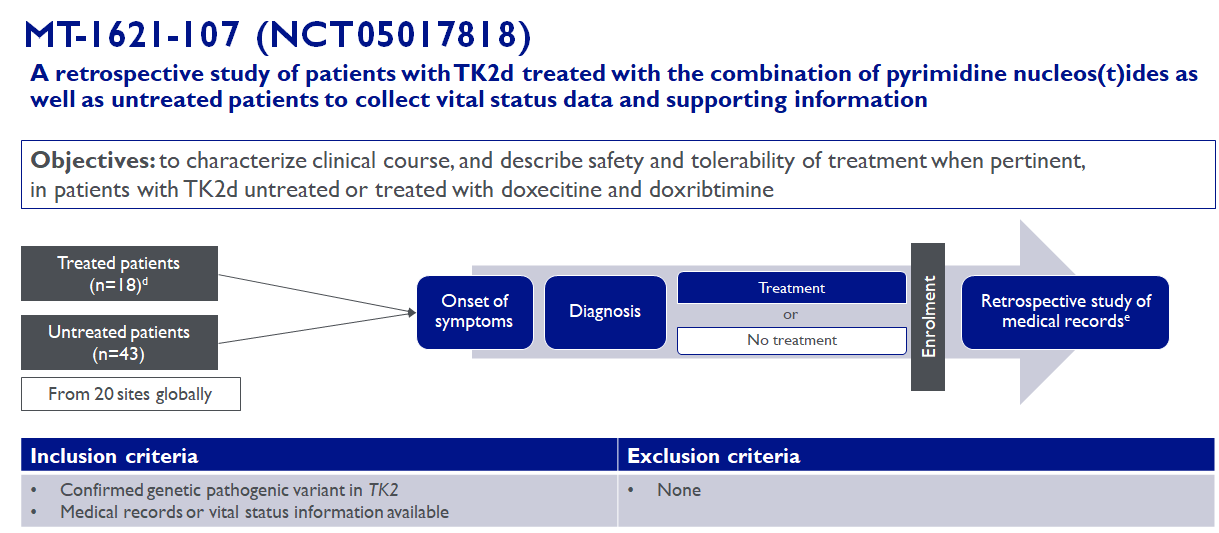


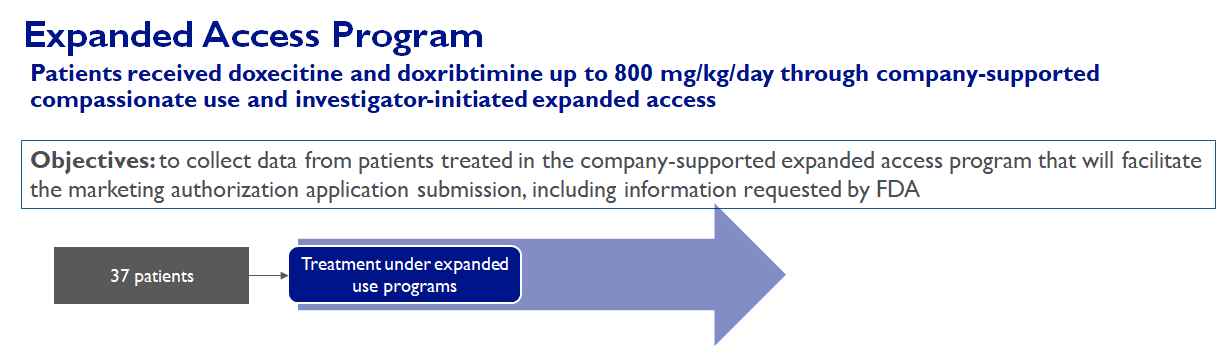


For the MT-1621-101 (NCT03701568) study, data for efficacy and safety pooled analyses were collected from 30 October 2018 to 28 March 2019 (with most recent patient visit for safety/efficacy assessment required to be between 1 June and 15 December 2018 (previously published).^1^ For the ongoing long-term follow-up study TK0102 (NCT03845712), interim data for efficacy and safety pooled analyses were collected from 10 July 2019 up to and including the data cut-off date on 15 March 2024 (data from some of the patients included in this study were published previously )^2^. For the MT-1621-107 (NCT05017818) study, data for efficacy and safety pooled analyses were collected from 22 September 2021 to 21 January 2022 (first report of these data). For the company-supported expanded access program, data cut-off date for inclusion in efficacy and safety pooled analyses was 1 March 2024 (first report of these data).

^a^dC and dT only: *n* = 32; any dCMP and dTMP: *n* = 6. ^b^Information extracted includes demographics; diagnosis and initial clinical presentations; clinical course, motor, function and ambulatory assessments; quality-of-life assessments; safety assessments; details of respiratory and feeding status; and any other assessments relevant to TK2d. ^c^Assessments include physical examination; 12-lead ECG; clinical laboratory tests; measurement of urine, blood and biomarker samples; motor and respiratory function assessments; CGI-I; PGI-I; INQoL questionnaire; and adverse events. ^d^Treatments were non-GMP dC and dT: *n* = 13; non-GMP dCMP and dTMP: *n* = 4; doxecitine and doxribtimine: *n* = 7 (groups not mutually exclusive). ^e^Information extracted includes vital status (age of last contact and/or age of death); demographics; family history; timing of motor milestones; details of respiratory and feeding support; CGI-S and CGI-I; treatment details and adverse events.

CGI = Clinical Global Impressions; CGI-I = CGI of Improvement; CGI-S = CGI of Severity; dC = deoxycytidine; dCMP = deoxycytidine monophosphate; dT = deoxythymidine; dTMP = deoxythymidine monophosphate; FDA = US Food and Drug Administration; GMP = good manufacturing practice; INQoL = Individualized Neuromuscular Quality of Life; PGI-I = Patient Global Impression of Improvement; TK2 = thymidine kinase; TK2d = thymidine kinase 2 deficiency.

**Supplementary Figure 2** **Survival analysis models**


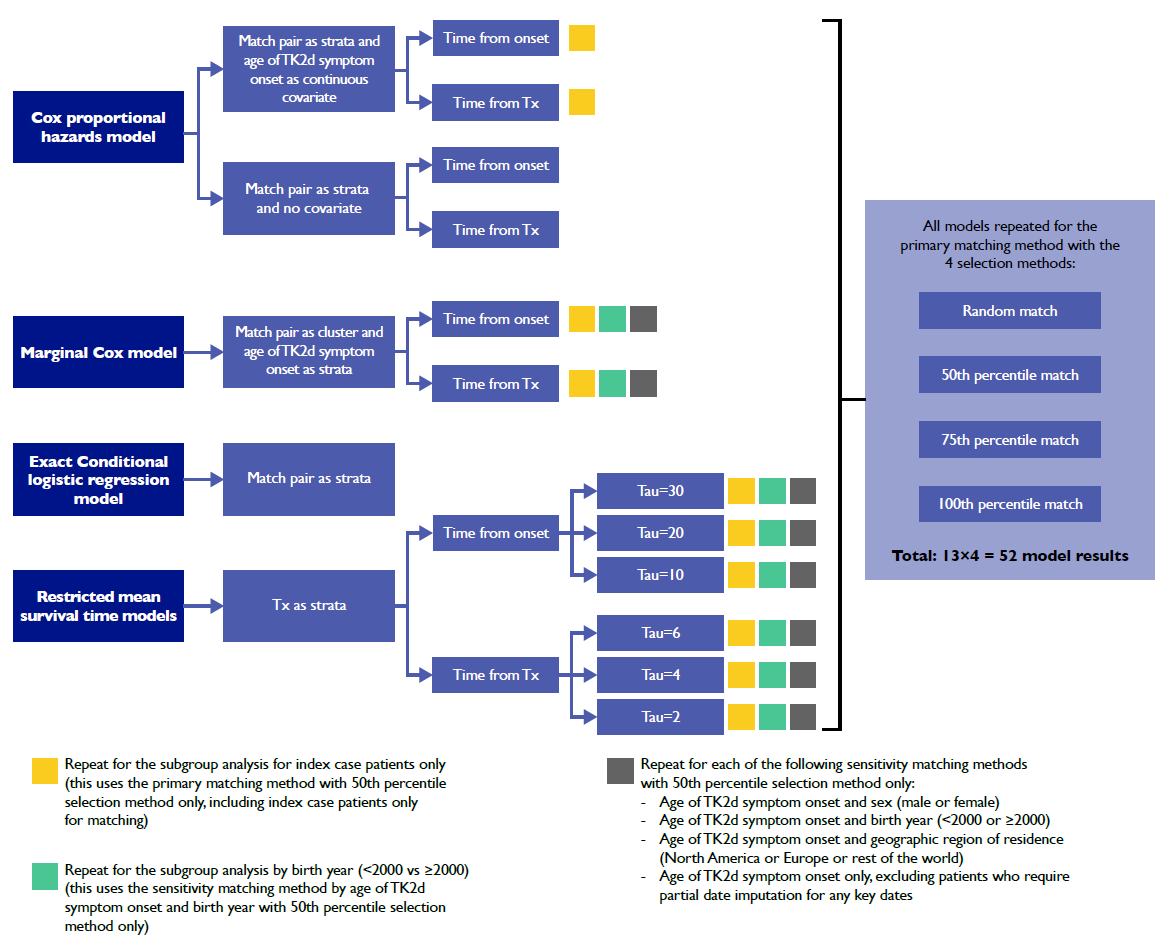


Tau refers to the cut-off time point used for each analysis.

TK2d = thymidine kinase 2 deficiency; Tx = treatment.

**Supplementary Figure 3** **Cox models forest plots for all age-of-symptom-onset subgroups and matching selection methods (from TK2d symptom onset and from treatment start)**


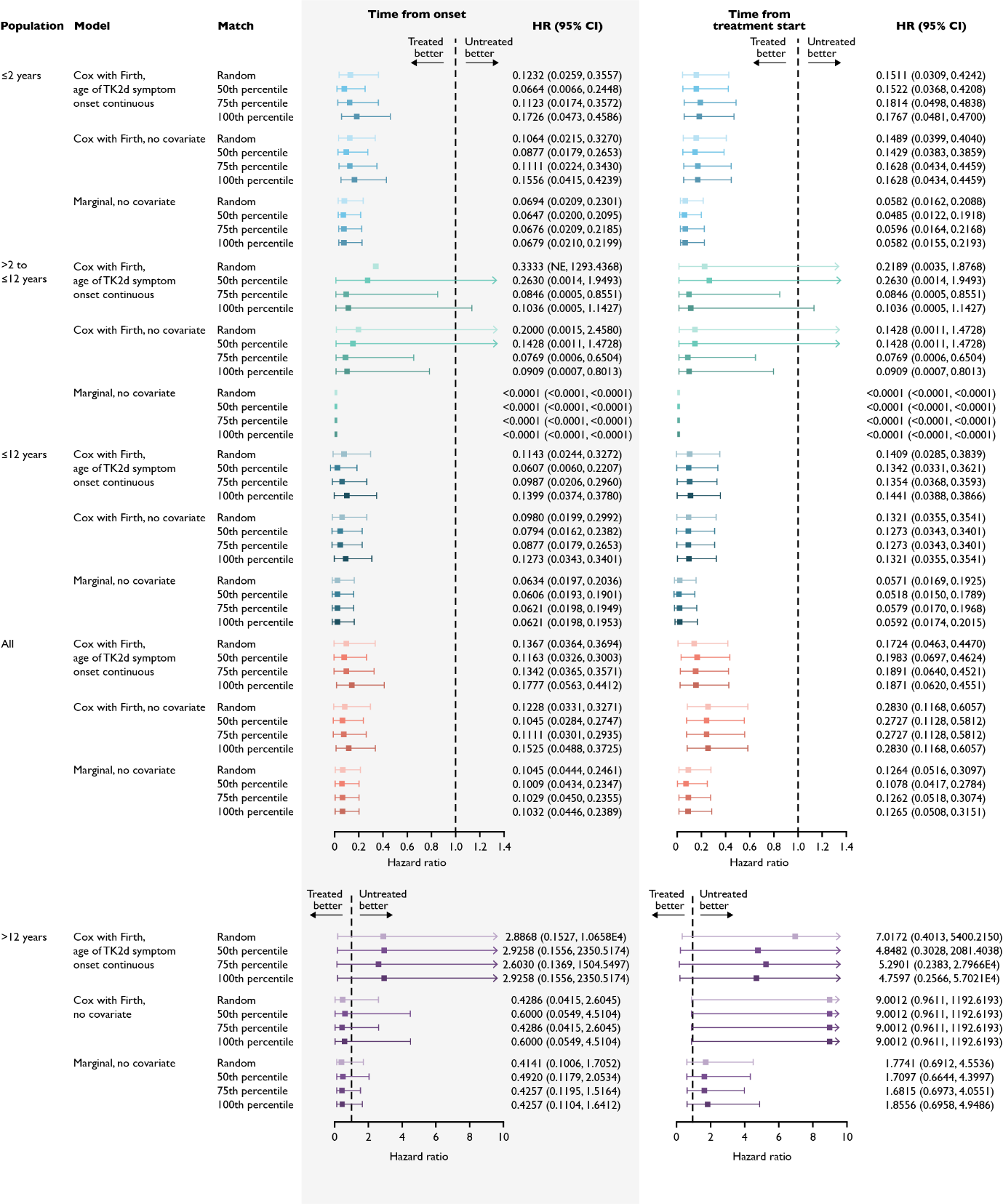


Number of patients included in each population: 56 treated patients and 69 untreated patients in the age-of-symptom-onset-≤2 years subgroup; 26 treated patients and 24 untreated patients in the age-of-symptom-onset->2 to ≤12 years subgroup; 82 treated patients and 93 untreated patients in the age-of-symptom-onset-≤12 years subgroup; 104 treated patients and 114 untreated patients in the overall population; 22 treated patients and 21 untreated patients in the age-of-symptom-onset->12 years subgroup.

CI = confidence interval; HR = hazard ratio; TK2d = thymidine kinase 2 deficiency.

**Supplementary Figure 4** **RMST forest plots for all age-of-symptom-onset subgroups and matching selection methods (from TK2d symptom onset and from treatment start)**


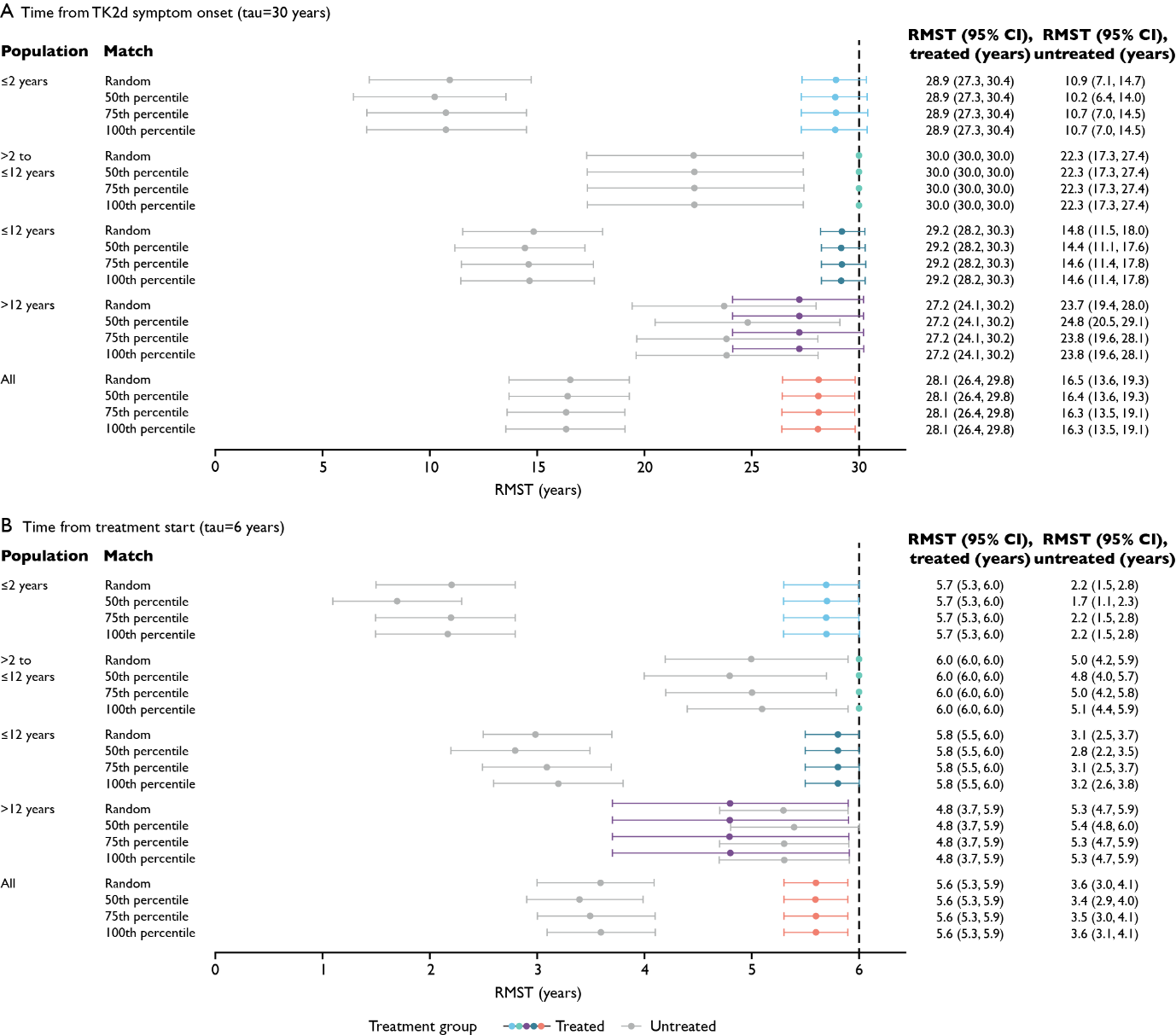


Tau refers to the cut-off time point used for each analysis.

Number of patients included in each population: 56 treated patients and 69 untreated patients in the age-of-symptom-onset-≤2 years subgroup; 26 treated patients and 24 untreated patients in the age-of-symptom-onset->2 to ≤12 years subgroup; 82 treated patients and 93 untreated patients in the age-of-symptom-onset-≤12 years subgroup; 22 treated patients and 21 untreated patients in the age-of-symptom-onset->12 years subgroup; 104 treated patients and 114 untreated patients in the overall population.

CI = confidence interval; RMST = restricted mean survival time; TK2d = thymidine kinase 2 deficiency.

**Supplementary Figure 5** **Summary of motor milestone loss and regain for age-of-symptom-onset-≤2-years and >2-to-≤12-years subgroups**

**
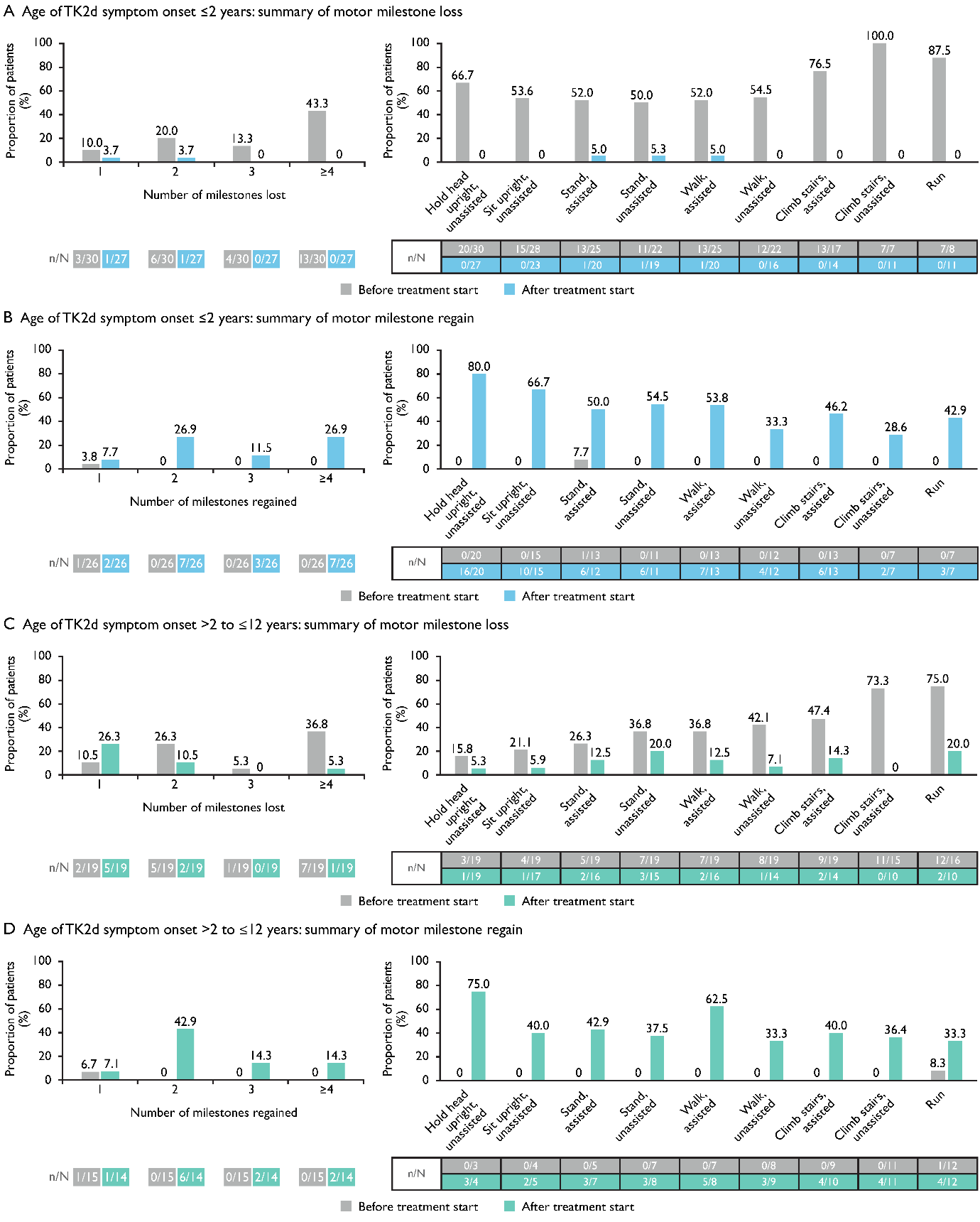
**

(A) Proportion of patients with age of symptom onset ≤2 years who lost developmental motor milestones before and after treatment start; (B) Proportion of patients with age of symptom onset ≤2 years who regained previously lost developmental motor milestones before and after treatment start; (C) Proportion of patients with age of symptom onset >2 to ≤12 years who lost developmental motor milestones before and after treatment start; (D) Proportion of patients with age of symptom onset >2 to ≤12 years who regained previously lost developmental motor milestones before and after treatment start.

TK2d = thymidine kinase 2 deficiency.

**Supplementary Table 1 Data contributing to the characterization of the TK2d disease course and efficacy and safety analyses**

| Data source | Population | Number of patients^a^ | Design | Data collection method | Data collection time points | Data contributing to course of disease evaluation |
| --- | --- | --- | --- | --- | --- | --- |
| **Untreated patient group^b^** | | | | | | |
| Updated-UPD (Includes patients from NCT03701568) | Patients with TK2d identified via published comprehensive literature reviews, case series and case reports, and with patient-level data and genetic confirmation of a *TK2* pathogenic variant, who were not treated with pyrimidine nucleos(t)ides | *N* = 113 | NA – all studies included irrespective of the study design | Literature review | NA – limited to data available in publications | Demographic and disease characteristics, survival, ventilatory support status |
| MT-1621-107  (NCT05017818) | Patients with genetically confirmed TK2d, who were not treated with pyrimidine nucleos(t)ides | *N* = 40 | Phase 2, non-interventional, multicentre medical chart review program to collect vital status data and related information on treated and untreated patients with TK2d from studies outside those conducted by the sponsor | Medical chart review | Longitudinal course of untreated patients | Demographic and disease characteristics, survival, motor milestones (acquisition, loss, regain, net gain), ventilatory support status, feeding support status |
| **Treated patient group** | | | | | | |
| MT-1621-101 (NCT03701568) | Patients with genetically confirmed TK2d treated with non-GMP dC and dT or dCMP and dTMP | *N* = 38 | Phase 2, multicentre, retrospective, non-interventional medical chart review | Medical chart review | Longitudinal, treatment course | Demographic and disease characteristics, survival, motor milestones (acquisition, loss, regain, net gain), ventilatory support status, feeding support status |
| TK0102 (NCT03845712) | Patients with genetically confirmed TK2d who previously participated in MT-1621‑101 and patients treated with dC and dT, dCMP and dTMP, or doxecitine and doxribtimine (GMP-grade dC and dT) who did not participate in MT-1621-101 | *N* = 12 | Phase 2, prospective, open-label treatment program of the efficacy and safety of doxecitine and doxribtimine in patients with TK2d | Data collected at screening visit for unique patients and followed prospectively  Medical chart review for the patients carried over from MT-1621-101 and followed prospectively | Longitudinal, treatment course | Demographic and disease characteristics, survival, motor milestones (acquisition, loss, regain, net gain), ventilatory support status, feeding support status |
| MT-1621-107 (NCT05017818) | Patients with genetically confirmed TK2d treated with chemical-grade dC and dT, dCMP and dTMP, or doxecitine and doxribtimine (GMP-grade dC and dT) outside of a study conducted by the sponsor | *N* = 17 | Phase 2, non-interventional, multicentre medical chart review program to collect vital status data and related information on treated and untreated patients with TK2d from studies outside those conducted by the sponsor | Medical chart review | Longitudinal course of untreated and treatment course | Demographic and disease characteristics, survival, motor milestones (acquisition, loss, regain, net gain), ventilatory support status, feeding support status |
| Company-supported Expanded access program | Paediatric and adult patients with TK2d with rapid disease progression and who are the most severely affected in accordance with eligibility criteria | *N* = 37 | Expanded access program | Data collected at screening and prospectively | Treatment course | Demographic and disease characteristics, survival |

^a^Only unique patients were included in analyses.

^b^Supplemental data collection for untreated patients from publications contributing to the database was performed to capture missing data on birth year and country of residence.

dC = deoxycytidine; dCMP = deoxycytidine monophosphate; dT = deoxythymidine; dTMP = deoxythymidine monophosphate; NA = not applicable; TK2d = thymidine kinase 2 deficiency; UPD = Untreated Patient Database.

**Supplementary Table 2** **Baseline demographics and characteristics for the age-of-symptom-onset-≤2-years and >2-to-≤12-years subgroups**

|  | **Patients with age of TK2d symptom onset ≤2 years** | | **Patients with age of TK2d symptom onset >2 to ≤12 years** | | |
| --- | --- | --- | --- | --- | --- |
|  | **Treated** | **Untreated** | **Treated** | **Untreated** | |
| ***N*** | 56 | 69 | 26 | 24 |  |
| **Sex, *n* (%)**  Male  Female | 31 (55.4)  25 (44.6) | 36 (52.2)  33 (47.8) | 15 (57.7)  11 (42.3) | 13 (54.2)  11 (45.8) | |
| **Race,^a^ *n* (%)**  White  Other  Not reported | 43 (76.8)  9 (16.1)  4 (7.1) | 15 (21.7)  2 (2.9)  52 (75.4) | 24 (92.3)  2 (7.7)  0 (0) | 9 (37.5)  0 (0)  15 (62.5) | |
| **Ethnicity, *n* (%)**  Hispanic or Latino  Not Hispanic or Latino  Not reported | 18 (32.1)  29 (51.8)  9 (16.1) | 7 (10.1)  10 (14.5)  52 (75.4) | 12 (46.2)  12 (46.2)  2 (7.7) | 5 (20.8)  4 (16.7)  15 (62.5) | |
| **Geographic region of residence,^a^ *n* (%)**  Europe  Rest of the world  Not reported | 18 (32.1)  38 (67.9)  0 (0) | 15 (21.7)  33 (47.8)  21 (30.4) | 9 (34.6)  17 (65.4)  0 (0) | 5 (20.8)  14 (58.3)  5 (20.8) | |
| **Age of TK2d symptom onset, years**  Median (min, max)  Q1, Q3 | 1.23 (0.01, 2.00)  0.92, 1.50 | 1.00 (0.00, 2.00)  0.60, 1.49 | 2.84 (2.03, 11.67)  2.42, 5.00 | 4.00 (2.49, 11.00)  3.00, 7.50 | |
| **Age at first treatment  (any treatment), years**  Median (min, max)  Q1, Q3 | 2.82 (0.69, 31.66)  1.72, 5.76 | NA  NA | 9.82 (2.78, 35.52)  6.79, 17.23 | NA  NA | |

^a^Owing to the ultra-rare nature of TK2d and the small number of patients, some details relating to race and country of residence were grouped for reporting purposes to minimize risk of patient identification.

min = minimum; max = maximum; NA = not applicable; Q1 = quartile 1; Q3 = quartile 3; TK2d = thymidine kinase 2 deficiency.

**Supplementary Table 3** **Baseline demographics and characteristics of the pooled safety population**

|  | **MT-1621-101, TK0102 and MT‑1621-107** | **MT-1621-101 and TK0102** |
| --- | --- | --- |
| ***N*** | 67 | 50 |
| **Sex, *n* (%)**  Male  Female | 38 (56.7)  29 (43.3) | 28 (56.0)  22 (44.0) |
| **Race,^a^ *n* (%)**  White  Other  Not reported | 61 (91.0)  5 (7.5)  1 (1.5) | 47 (94.0)  3 (6.0)  0 (0) |
| **Ethnicity, *n* (%)**  Hispanic or Latino  Not Hispanic or Latino  Not reported  Unknown | 15 (22.4)  49 (73.1)  1 (1.5)  2 (3.0) | 14 (28.0)  36 (72.0)  0 (0)  0 (0) |
| **Geographic region of residence,^a^ *n* (%)**  Europe  Rest of the world | 34 (50.7)  33 (49.3) | 27 (54.0)  23 (46.0) |
| **Age of TK2d symptom onset, years**  Median (min, max)  Q1, Q3 | 2.05 (0.0, 60.3)  1.21, 12.36 | 2.37 (0.3, 60.3)  1.34, 6.09 |
| **Age at first treatment (any treatment), years**  Median (min, max)  Q1, Q3 | 6.79 (0.7, 74.0)  2.52, 28.97 | 7.63 (0.7, 74.0)  2.55, 26.59 |

^a^Owing to the ultra-rare nature of TK2d and the small number of patients, some details relating to race and country of residence were grouped for reporting purposes to minimize risk of patient identification.

Data on safety were summarized in the ISS pooled safety population. ISS pooled safety population includes patients with TK2d who received at least one dose of pyrimidine nucleos(t)ide therapy in the studies MT-1621-101, TK0102 and MT-1621-107 (data from MT-1621-107 included when available).

min = minimum; max = maximum; Q1 = quartile 1; Q3 = quartile 3; TK2d = thymidine kinase 2 deficiency.

**Supplementary Table 4 Summary of study drug administration, including duration and delay to initiation for age-of- symptom-onset-≤2-years and >2-to-≤12-years subgroups**

|  | **Treated** | |
| --- | --- | --- |
|  | **Patients with age of TK2d symptom onset ≤2 years** | **Patients with age of TK2d symptom onset >2 to ≤12years** |
| ***N*** | 56 | 26 |
| **Treatment duration (months)**  Mean (SD)  Median (min, max)  Q1, Q3 | 47.43 (41.69)  30.82 (0.04, 133.22)  10.30, 83.77 | 60.28 (40.96)  70.38 (1.78, 157.07)  18.94, 77.24 |
| **Treatment duration category, *n* (%)**  At least one dose  2 days–<1 month  1 month–<3 months  3–<6 months  6–<12 months  12–<15 months  15–<18 months  18 months–<2 years  2–<5 years  ≥5 years | 56 (100)  1 (1.8)  2 (3.6)  6 (10.7)  5 (8.9)  0 (0)  5 (8.9)  6 (10.7)  11 (19.6)  19 (33.9) | 26 (100)  0 (0)  1 (3.8)  1 (3.8)  2 (7.7)  1 (3.8)  1 (3.8)  1 (3.8)  5 (19.2)  14 (53.8) |
| **Time from TK2d symptom onset to start of treatment, *n* (%)**  ≤6 months  >6 months–≤1 year  >1 year–≤5 years  >5–≤10 years  >10 years | 11 (19.6)  12 (21.4)  19 (33.9)  6 (10.7)  8 (14.3) | 0 (0)  1 (3.8)  10 (38.5)  7 (26.9)  8 (30.8) |

min = minimum; max = maximum; Q1 = quartile 1; Q3 = quartile 3; SD = standard deviation;
TK2d = thymidine kinase 2 deficiency.

**Supplementary Table 5** **Summary of patient survival for age-of-symptom-onset-≤2-years and >2-to-≤12-years subgroups**

|  | **Patients with age of TK2d symptom onset ≤2 years** | | **Patients with age of TK2d symptom onset >2 to ≤12 years** | |
| --- | --- | --- | --- | --- |
|  | **Treated** | **Untreated** | **Treated** | **Untreated** |
| ***N*** | 56 | 69 | 26 | 24 |
| **Reported death, *n* (%)** | 3 (5.4) | 47 (68.1) | 0 (0) | 6 (25.0) |
| **Age at death, years**  Mean (SD)  Median (min, max)  Q1, Q3 | *n* = 3  11.27 (17.75)  1.11 (0.94, 31.77)  0.94, 31.77 | *n* = 47  3.47 (4.54)  2.00 (0.83, 29.94)  1.54, 3.50 | *n* = 0  NA  NA  NA | *n* = 6  12.96 (11.12)  10.25 (3.00, 33.50)  4.75, 16.00 |
| **No reported death, *n* (%)**  Last known age alive  Missing age alive | 53 (94.6)  53 (100)  0 (0) | 22 (31.9)  22 (100)  0 (0) | 26 (100)  26 (100)  0 (0) | 18 (75.0)  18 (100)  0 (0) |
| **Age last known alive, years**  Mean (SD)  Median (min, max)  Q1, Q3 | *n* = 53  9.99 (8.30)  7.65 (1.1, 34.5)  4.79, 12.05 | *n* = 22  11.43 (12.76)  5.35 (0.8, 53.0)  4.00, 14.00 | *n* = 26  17.53 (9.56)  14.34 (4.8, 41.0)  9.86, 21.98 | *n* = 18  19.58 (11.72)  17.50 (4.3, 50.0)  10.00, 21.00 |

min = minimum; max = maximum; NA = not applicable; Q1 = quartile 1; Q3 = quartile 3; SD = standard deviation; TK2d = thymidine kinase 2 deficiency.

**Supplementary Table 6 Summary of ventilatory and enteral feeding tube support for treated patients in age-of-symptom-onset-≤2-years and >2-to-≤12-years subgroups**

|  | **Patients with age of TK2d symptom onset  ≤2 years  (*N* = 56)** | | **Patients with age of TK2d symptom onset  >2 to ≤12 years  (*N* = 26)** | |
| --- | --- | --- | --- | --- |
|  | **Before treatment** | **After  treatment** | **Before treatment** | **After  treatment** |
| **Summary of ventilatory support** |  |  |  |  |
| Initiated ventilatory support, *n/N* (%) | 21/56 (37.5) | 2/13^a^ (15.4) | 10/26 (38.5) | 2/9^a^ (22.2) |
| Discontinued ventilatory support, *n/N* (%) | 0/21^b^ (0) | 0/2 (0) | 0/10^b^ (0) | 1/2 (50.0) |
| Hours of ventilatory support per day (last observation) | | | | |
| *n* | 18 | 10 | 10 | 7 |
| Median (min, max) | 12.5 (8.0, 24.0) | 10.5 (0, 24.0) | 8.0 (8.0, 24.0) | 8.0 (0.0, 24.0) |
| Q1, Q3 | 10.0, 24.0 | 0.0, 18.0 | 8.0, 12.0 | 0.0, 10.0 |
| No ventilatory support data collected, *n* (%) | 22 (39.3) | | 7 (26.9) | |
| **Summary of enteral feeding tube support** | |  |  |  |
| Enteral feeding tube inserted, *n/N* (%) | 16/56 (28.6) | 3/18^a^ (16.7) | 4/26 (15.4) | 1/15^a^ (6.7) |
| Reason for tube insertion,^d^ *n/N* (%) | | | | |
| Supplemental oral intake | 5/16 (31.3) | 2/3 (66.7) | 3/4 (75.0) | 1/1 (100) |
| Dysphagia | 13/16 (81.3) | 1/3 (33.3) | 4/4 (100) | 1/1 (100) |
| Other | 3/16 (18.8) | 1/3 (33.3) | 0/4 (0) | 0/1 (0) |
| Enteral feeding tube removed, *n/N* (%) | 1/16^b^ (6.3) | 1/3^c^ (33.3) | 0/4^b^ (0) | 1/1^c^ (100) |
| Reason for tube removal,^d^ *n/N* (%) | | | | |
| Improvement not otherwise specified | 0/1 (0) | 1/1 (100) | 0 (0) | 0/1 (0) |
| Improvement dysphagia | 0/1 (0) | 0/1 (0) | 0 (0) | 0/1 (0) |
| Improved oral feeding | 1/1 (100) | 0/1 (0) | 0 (0) | 0/1 (0) |
| Other | 1/1 (100) | 0/1 (0) | 0 (0) | 0/1 (0) |
| Adverse device event | 0/1 (0) | 0/1 (0) | 0 (0) | 1/1 (100) |
| No enteral feeding tube support data collected, *n* (%) | 23 (41.1) | | 7 (26.9) | |

^a^N is patients with available data not using support before treatment initiation who were at risk of starting support after treatment initiation.

^b^N is patients using support before treatment initiation who were at risk of discontinuing support.

^c^N is patients using support at any time after treatment initiation who were at risk of discontinuing support.

^d^Patients could be counted in more than one category for reasons for tube insertion and tube removal.

min = minimum; max = maximum; Q1 = quartile 1; Q3 = quartile 3; TK2d = thymidine kinase 2 deficiency.

**Supplementary Table 7** **Summary of TEAEs by system organ class and preferred term in at least 10% of patients in the pooled safety population**

| **Patients with TEAEs by system organ class and preferred term, *n* (%)** | **MT-1621-101 and TK0102 (*N* = 50)** | **MT-1621-101, TK0102 and MT‑1621-107 (*N* = 67)** |
| --- | --- | --- |
| **Gastrointestinal disorders** | 46 (92.0) | – |
| Diarrhoea | 43 (86.0) | – |
| Vomiting | 14 (28.0) | – |
| Abdominal pain | 10 (20.0) | – |
| Dysphagia | 8 (16.0) | – |
| Gastro-oesophageal reflux disease | 6 (12.0) | – |
| Abdominal pain upper | 5 (10.0) | – |
| Constipation | 5 (10.0) | – |
| **General disorders and administration-site conditions** | 29 (58.0) | – |
| Pyrexia | 20 (40.0) | – |
| Influenza-like illness | 6 (12.0) | – |
| **Infections and infestations** | 43 (86.0) | – |
| Upper respiratory tract infection | 19 (38.0) | – |
| COVID-19 | 18 (36.0) | – |
| Pneumonia | 10 (20.0) | – |
| Respiratory tract infection | 10 (20.0) | – |
| Gastroenteritis | 9 (18.0) | – |
| Influenza | 8 (16.0) | – |
| Ear infection | 7 (14.0) | – |
| Nasopharyngitis | 5 (10.0) | – |
| Urinary tract infection | 5 (10.0) | – |
| **Injury, poisoning and procedural complications** | 20 (40.0) | – |
| Femur fracture | 5 (10.0) | – |
| **Investigations** | 35 (70.0) | – |
| ALT increased | 14 (28.0) | – |
| AST increased | 11 (22.0) | – |
| Blood creatine phosphokinase increased | 10 (20.0) | – |
| Anion gap increased | 7 (14.0) | – |
| Blood lactic acid increased | 7 (14.0) | – |
| Basophil count increased | 5 (10.0) | – |
| γ-glutamyl transferase increased | 5 (10.0) | – |
| Platelet count increased | 5 (10.0) | – |
| **Musculoskeletal and connective tissue disorders** | 22 (44.0) | – |
| Muscular weakness | 7 (14.0) | – |
| Arthralgia | 6 (12.0) | – |
| Back pain | 5 (10.0) | – |
| Pain in extremity | 5 (10.0) | – |
| **Nervous system disorders** | 22 (44.0) | – |
| Headache | 13 (26.0) | – |
| **Respiratory, thoracic and mediastinal disorders** | 34 (68.0) | – |
| Rhinorrhoea | 15 (30.0) | – |
| Cough | 11 (22.0) | – |
| Oropharyngeal pain | 8 (16.0) | – |
| Acute respiratory failure | 5 (10.0) | – |
| Dyspnoea | 5 (10.0) | – |
| **Skin and subcutaneous tissue disorders** | 17 (34.0) | – |
| Rash | 7 (14.0) | – |

All adverse event terms were coded to Medical Dictionary for Regulatory Activities version 26.0.

ALT = alanine aminotransferase; AST = aspartate aminotransferase; TEAE = treatment-emergent adverse event.

**Supplementary Table 8 Summary of TEAEs leading to study drug discontinuation and dose reduction in the pooled safety population**

|  | **MT-1621-101, TK0102 and MT‑1621-107 (*N* = 67)** |
| --- | --- |
| **Patients with TEAE leading to study drug discontinuation, *n* (%)** | 9 (13.4) |
| **Patients with TEAEs leading to study drug discontinuation by system organ class and preferred term, n (%)** | |
| Congenital, familial and genetic disorders | 1 (1.5) |
| Mitochondrial DNA depletion | 1 (1.5) |
| Gastrointestinal disorders | 4 (6.0) |
| Diarrhoea | 2 (3.0) |
| Constipation | 1 (1.5) |
| Nausea | 1 (1.5) |
| Vomiting | 1 (1.5) |
| General disorders and administration-site conditions | 1 (1.5) |
| Death | 1 (1.5) |
| Investigations | 2 (3.0) |
| γ-glutamyl transferase increased | 1 (1.5) |
| Hepatic enzyme increased | 1 (1.5) |
| Nervous system disorders | 1 (1.5) |
| Seizure | 1 (1.5) |
| **Patients with TEAE leading to dose reduction, n (%)** | 16 (23.9) |
| **Patients with TEAEs leading to dose reduction by system organ class and preferred term, n (%)** | |
| Gastrointestinal disorders | 15 (22.4) |
| Diarrhoea | 14 (20.9) |
| Abdominal pain upper | 2 (3.0) |
| Frequent bowel movements | 2 (3.0) |
| Abdominal pain | 1 (1.5) |
| Vomiting | 1 (1.5) |
| Infection and infestations | 1 (1.5) |
| COVID-19 | 1 (1.5) |
| Investigations | 3 (4.5) |
| ALT increased | 1 (1.5) |
| AST increased | 1 (1.5) |
| Stool pH decreased | 1 (1.5) |
| Weight decreased | 1 (1.5) |
| Skin and subcutaneous tissue disorders | 1 (1.5) |
| Erythema | 1 (1.5) |

All adverse event terms were coded to Medical Dictionary for Regulatory Activities version 26.0.

ALT = alanine aminotransferase; AST = aspartate aminotransferase; TEAE = treatment-emergent adverse event.

**Supplementary Table 9 Summary of TEAEs by system organ class, preferred term and CTCAE grade in >10% of patients in the pooled safety population**

|  | **All formulation (*N* = 50)** | | | | |
| --- | --- | --- | --- | --- | --- |
|  | CTCAE grade | | | | |
|  | 1 n (%) | 2 n (%) | 3 n (%) | 4 n (%) | 5 n (%) |
| Patients with at least one TEAE | 5 (10.0) | 16 (32.0) | 21 (42.0) | 7 (14.0) | 1 (2.0) |
| Gastrointestinal disorders | 15 (30.0) | 17 (34.0) | 13 (26.0) | 1 (2.0) | 0 |
| Diarrhoea | 24 (48.0) | 12 (24.0) | 7 (14.0) | 0 | 0 |
| Vomiting | 9 (18.0) | 1 (2.0) | 4 (8.0) | 0 | 0 |
| General disorders and administration-site conditions | 14 (28.0) | 4 (8.0) | 10 (20.0) | 0 | 1 (2.0) |
| Pyrexia | 10 (20.0) | 2 (4.0) | 8 (16.0) | 0 | 0 |
| Influenza like illness | 6 (12.0) | 0 | 0 | 0 | 0 |
| Infections and infestations | 14 (28.0) | 16 (32.0) | 11 (22.0) | 2 (4.0) | 0 |
| COVID-19 | 10 (20.0) | 6 (12.0) | 2 (4.0) | 0 | 0 |
| Gastroenteritis | 1 (2.0) | 8 (16.0) | 0 | 0 | 0 |
| Upper respiratory tract infection | 8 (16.0) | 9 (18.0) | 2 (4.0) | 0 | 0 |
| Investigations | 16 (32.0) | 4 (8.0) | 15 (30.0) | 0 | 0 |
| ALT increased | 10 (20.0) | 2 (4.0) | 2 (4.0) | 0 | 0 |
| AST increased | 8 (16.0) | 1 (2.0) | 2 (4.0) | 0 | 0 |
| Blood creatine phosphokinase increased | 7 (14.0) | 2 (4.0) | 1 (2.0) | 0 | 0 |
| Musculoskeletal and connective tissue disorders | 13 (26.0) | 7 (14.0) | 2 (4.0) | 0 | 0 |
| Arthralgia | 6 (12.0) | 0 | 0 | 0 | 0 |
| Nervous system disorders | 12 (24.0) | 4 (8.0) | 6 (12.0) | 0 | 0 |
| Headache | 8 (16.0) | 2 (4.0) | 3 (6.0) | 0 | 0 |
| Respiratory, thoracic and mediastinal disorders | 11 (22.0) | 7 (14.0) | 11 (22.0) | 5 (10.0) | 0 |
| Cough | 2 (4.0) | 1 (2.0) | 8 (16.0) | 0 | 0 |
| Rhinorrhoea | 11 (22.0) | 1 (2.0) | 3 (6.0) | 0 | 0 |
| Skin and subcutaneous tissue disorders | 12 (24.0) | 4 (8.0) | 1 (2.0) | 0 | 0 |
| Rash | 7 (14.0) | 0 | 0 | 0 | 0 |

All adverse event terms were coded to Medical Dictionary for Regulatory Activities version 26.0.

ALT = alanine aminotransferase; AST = aspartate aminotransferase; CTCAE = Common Terminology Criteria for Adverse Events; TEAE = treatment-emergent adverse event.

# References

1. Dominguez-Gonzalez C, Chiang C, Colson AO*, et al*. Pyrimidine Nucleos(t)ide Therapy in Patients With Thymidine Kinase 2 Deficiency: A Multicenter Retrospective Chart Review Study. *Neurology*. Sep 23 2025;105(6):e213908. doi:10.1212/WNL.0000000000213908

2. Hernandez-Voth A, Sayas Catalan J, Corral Blanco M*, et al*. Deoxynucleoside therapy for respiratory involvement in adult patients with thymidine kinase 2-deficient myopathy. *BMJ Open Respir Res*. Nov 2020;7(1)doi:10.1136/bmjresp-2020-000774

**SAS codes and R scripts for survival analyses**

**Figure 2** Direct adjustment survival curves and RMSTs (from TK2d symptom onset and from treatment start; 50th-percentile matching estimated from marginal Cox models)

**SAS procedure for Direct Adjustment Curves:** proc phreg data=dataset covs(aggregate) ;

class trt (ref='0') strata2; */*for 50^th^ percentile matching*/*

model aval*cnsr(1)=trt/ties=breslow type3(all) risklimits=PL maxiter = 1000;

id strata2;

strata agegroup ;

hazardratio 'Marginal Model Analysis ' trt / diff=ref;

baseline covariates= dataset out=output-dataset survival=_all_/diradj group=trt;

run;

**SAS Procedure for REMST:**

proc lifetest data=dataset method=km rmst(tau=30) ; */* tau is 30 from time from symptom onset and tau =6 for time from treatment start*/*

by catn ;

time aval*cnsr(1);

strata trtn /diff = all notest;

test trtn;

ods output rmst = output rmstdiff = output_diff;

run;

**Supplementary Figure 3** Cox models forest plots for all age-of-symptom-onset subgroups and matching selection methods (from TK2d symptom onset and from treatment start)

**SAS procedure for Marginal model:**

proc phreg data=tte2 covs(aggregate) ;

class trt (ref='0') strata/*Random,100/75/50*/ ;

model aval*cnsr(1)=trt/ties=breslow type3(all) risklimits=PL maxiter = 1000;

id strata;

strata agegroup;

hazardratio 'Marginal Model Analysis ' trt / diff=ref;

ods output hazardratios=output modelanova = output1;

run;

**SAS procedure for Cox PH model:**

proc phreg data= dataset;

class;

model aval*cnsr(1)=trt ageogr2 RL ties=breslow type3(all) risklimits=both maxiter = 100000 FIRTH /*option to be removed if without firth correction*/;

ods output globaltests = output1 modelanova = output2 parameterestimates = output3 ConvergenceStatus = output4 ;

strata ;

run;

**Forest Plot in R:**

library(tidyr)

library(dplyr)

library(stringr)

library(ggplot2)

library(haven)

library(patchwork)

g_frst_hro <- read_sas("/link/dataset.sas7bdat")

X_WIDTH_MAX <- max(c(g_frst_hro$HR, g_frst_hro$HR_TRT))

data_plot <- g_frst_hro %>%

#format population and model to show the text for only the first row (also >2 and <=12 Years need to be on two rows to make space for the figure)

group_by(PART, PAGE, POPULATION, MODEL) %>% mutate(MODEL_P = ifelse(row_number()==1, MODEL, "")) %>% ungroup() %>%

group_by(PART, PAGE, POPULATION) %>% mutate(POP_P = case_when((POPULATION=="> 2 and <= 12 Years" & row_number()==1) ~ ">2 and",

(POPULATION=="> 2 and <= 12 Years" & row_number()==2) ~ "<=12 Years",

row_number()==1 ~ POPULATION,

TRUE ~ ""),

POP_P = ifelse(POP_P=="0-12", "<=12 Years", POP_P),

#formatting: to be consistent with other age groups

POP_P = gsub("= ", "=", POP_P),

POP_P = gsub("> ", ">", POP_P)) %>% ungroup() %>%

#formatting: remove spaces after "=" and ">"

group_by(PART, PAGE) %>% mutate(MODID_P = n():1) %>% ungroup() %>%

#Numeric Model ID for plotting purpose

#fix display of large numbers

mutate(tmp = ifelse(is.na(CI_H) | CI_H<10000, NA, str_replace_all(format(CI_H, scientific = T, digits = 5), "e\\+(0|)", "E")),

HRCI = ifelse(is.na(tmp), HRCI, str_replace_all(HRCI, ", .+", paste0(", ", tmp, ")"))),

tmp = NULL) %>%

mutate(tmp = ifelse(is.na(CI_HTRT) | CI_HTRT<10000, NA, str_replace_all(format(CI_HTRT, scientific = T, digits = 5), "e\\+(0|)", "E")),

HRCI_TRT = ifelse(is.na(tmp), HRCI_TRT, str_replace_all(HRCI_TRT, ", .+", paste0(", ", tmp, ")"))),

tmp = NULL) %>%

#cut CI upper bound at X_WIDTH_MAX+0.6 (>12 subgroup) or 1.35 (other) for plotting and will use an arrow to represent

mutate(CI_H_P = ifelse(POPULATION=="> 12 Years", ifelse(CI_H>X_WIDTH_MAX+0.6, X_WIDTH_MAX+0.6, CI_H), ifelse(CI_H>1.4, 1.35, CI_H)),

CI_HT_P = ifelse(POPULATION=="> 12 Years", ifelse(CI_HTRT>X_WIDTH_MAX+0.6, X_WIDTH_MAX+0.6, CI_HTRT), ifelse(CI_H>1.4, 1.35, CI_HTRT))) %>%

as.data.frame()

data_annotate <- data_plot %>%

group_by(PART, PAGE) %>%

#For each PART and PAGE, add two rows at the top for annotation purpose

group_modify(~add_row(.x, POP_P="", MODEL_P="", METHOD="", HRCI="", HRCI_TRT="", MODID_P=nrow(.x)+1, .before=0)) %>%

group_modify(~add_row(.x, POP_P="Population", MODEL_P="Model", METHOD="Match", HRCI="HR (95% CI)", HRCI_TRT="HR (95% CI)", MODID_P=nrow(.x)+1, .before=0)) %>%

as.data.frame()

```

```{r plotfun}

add_background <- function(p, bg_xmin=0, bg_xmax=0.1){

#function to add bands of background color

#p: a ggplot object

#bg_xmin: background rectangle xmin coordinate

#bg_xmax: background rectangle xmax coordinate

N <- floor(nrow(p$data)/4)

#one rectangle per 4 rows (corresponding to 4 matching selection methods)

bg_data <- data.frame(MIN = seq(from=0.5, by=4, length.out=N),

MAX = seq(from=4.5, by=4, length.out=N),

FILL = rep(c("snow3", "snow2", "snow"), length.out=N))

p_out <- p

for(i in 1:N){

p_out <- p_out +

geom_rect(xmin = bg_xmin, xmax = bg_xmax, ymin = bg_data$MIN[i], ymax = bg_data$MAX[i], fill = bg_data$FILL[i], color = NA)

}

return(p_out)

}

gg_column_text <- function(a_data, lab_name, bold_str, text_size=2.9){

#function to create a column of annotation text for the forestplot

#a_data: annotation data

#lab_name: the name of the column to be selected from a_data to be plotted

#bold_str: title (a string) for the column that will be bolded

#text_size: size of the annotation text

p_out <- add_background(ggplot(a_data)) +

geom_text(aes_string(x = 0, y = "MODID_P", label = lab_name), hjust = 0,

fontface = ifelse(a_data[,lab_name] == bold_str, "bold", "plain"), size=text_size) +

scale_x_continuous(limits = c(0, 0.1), expand = c(0, 0)) +

theme_void(base_size = 8)

return(p_out)

}

gg_column_frst <- function(p_data, x, y, xmin, xmax, xlab_str, x_breaks, x_range = NULL, errorbar_width = 0.15){

#function to create the column of 'forest' (HR and CI)

#p_data: plotting data (need to at least contain HR and CI)

#x, y: column names for (x, y) coordinates of the points representing HR (x should be HR, y should be Model)

#xmin, xmax: column names for CI lower bound and upper bound values for errorbar x coordinates

#xlab_str: name (a string) for x axis label

#x_breaks: to be passed to ggplot2::scale_x_continuous(breaks =)

#x_range: limits of x axis (vector of length 2), if NULL, will be set to (0, maximum of x_breaks)

if(is.null(x_range)){

x_range <- c(0, max(x_breaks))

}

#data for plotting the right arrow for CIs that were cutoff at X_WIDTH_MAX+0.6 (page 3 of any part) or 1.35 (other pages)

if(unique(p_data$PAGE)==3){

data_arrow <- p_data[p_data[, xmax]==X_WIDTH_MAX+0.6, ]

data_arrow[, paste0(xmax, "_END")] <- data_arrow[,xmax]+0.05

}else{

data_arrow <- p_data[p_data[, xmax]==1.35, ]

data_arrow[, xmax] <- data_arrow[, xmax]-0.02

data_arrow[, paste0(xmax, "_END")] <- data_arrow[, xmax]+0.03

}

p_out <- add_background(ggplot(p_data, aes_string(x = x, y = y, color = "METHOD")), bg_xmin = x_range[1], bg_xmax = x_range[2]) +

geom_point(shape=15, size=1.5) +

geom_errorbar(aes_string(xmin=xmin, xmax=xmax), width = errorbar_width) +

geom_vline(xintercept = 1, linetype = "dashed") +

geom_segment(data=data_arrow, aes_string(x = xmax, xend=paste0(xmax, "_END"), y = "MODID_P", yend = "MODID_P"), arrow = arrow(length=unit(0.15, "cm"))) +

xlab(xlab_str) +

scale_color_manual(values = c("Random"="skyblue", "50th Percentile"="royalblue1", "75th Percentile"="royalblue3", "100th Percentile"="royalblue4")) +

scale_x_continuous(breaks = x_breaks, limits = x_range, expand = c(0, 0)) +

coord_cartesian(ylim=c(1, nrow(p_data) + 2)) +

theme_classic(base_size = 8) +

theme(axis.line.y = element_blank(),

axis.ticks.y= element_blank(),

axis.text.y= element_blank(),

axis.title.y= element_blank(),

legend.position = "none",

plot.margin = margin(l=0,r=0))

return(p_out)

}

add_direction <- function(p, x_trt, x_untrt, y_top, arrow_len){

#function to add the text and arrows to indicate directions for treated better vs. untreated better

#p: a ggplot object

#x_trt: x coordinate for the text "Treated better"

#x_untrt: x coordinate for the text "Untreated better"

#y_top: y coordinate for the top row of the text

#arrow_len: length of the arrow

p_out <- p +

annotate("text", x = x_trt, y = y_top, label = "Treated", size = 2, hjust=1) +

annotate("text", x = x_trt, y = y_top-0.45, label = "better", size = 2, hjust=1) +

annotate("segment", x = x_trt, xend = x_trt - arrow_len, y = y_top-0.9, yend = y_top-0.9, arrow=arrow(length=unit(0.1, "cm"))) +

annotate("text", x = x_untrt, y = y_top, label = "Untreated", size = 2, hjust=0) +

annotate("text", x = x_untrt, y = y_top-0.45, label = "better", size = 2, hjust=0) +

annotate("segment", x=x_untrt, xend = x_untrt + arrow_len, y = y_top-0.9, yend = y_top-0.9, arrow=arrow(length=unit(0.1, "cm")))

return(p_out)

}

p_pop <- data_annotate %>% filter(PART==1, PAGE==1) %>% gg_column_text(lab_name = "POP_P", bold_str = "Population")

p_mod <- data_annotate %>% filter(PART==1, PAGE==1) %>% gg_column_text(lab_name = "MODEL_P", bold_str = "Model")

p_mat <- data_annotate %>% filter(PART==1, PAGE==1) %>% gg_column_text(lab_name = "METHOD", bold_str = "Match")

p_onset <- data_plot %>% filter(PART==1, PAGE==1) %>%

gg_column_frst(x="HR", y="MODID_P", xmin = "CI_L", xmax="CI_H_P", xlab_str = "Time from Onset", x_breaks = seq(0, 1.4, by=0.2)) %>%

add_direction(x_trt = 0.9, x_untrt = 1.1, arrow_len =0.2, y_top = 25.7)

p_hrci <- data_annotate %>% filter(PART==1, PAGE==1) %>% gg_column_text(lab_name = "HRCI", bold_str = "HR (95% CI)")

p_trt <- data_plot %>% filter(PART==1, PAGE==1) %>%

gg_column_frst(x="HR_TRT", y="MODID_P", xmin = "CI_LTRT", xmax="CI_HT_P", xlab_str = "Time from Treatment", x_breaks = seq(0, 1.4, by=0.2)) %>%

add_direction(x_trt = 0.9, x_untrt = 1.1, arrow_len =0.2, y_top = 25.7)

p_hrci_t <- data_annotate %>% filter(PART==1, PAGE==1) %>% gg_column_text(lab_name = "HRCI_TRT", bold_str = "HR (95% CI)")

#combine columns

layout <- c(

area(t = 0, l = 0, b = 30, r = 3),

area(t = 0, l = 4, b = 30, r = 9),

area(t = 0, l = 10, b = 30, r = 13),

area(t = 0, l = 14, b = 30, r = 20),

area(t = 0, l = 21, b = 30, r = 26),

area(t = 0, l = 27, b = 30, r = 33),

area(t = 0, l = 34, b = 30, r = 39)

)

p_pop + p_mod + p_mat + p_onset + p_hrci + p_trt + p_hrci_t + plot_layout(design = layout)

**Supplementary Figure 4** RMST forest plots for all age-of-symptom-onset subgroups and matching selection methods (from TK2d symptom onset and from treatment start)

**SAS procedure for REMST:**

proc lifetest data=tte1 rmst (tau=30) method=km alpha=0.05;

by catn;

time aval*cnsr(1);

strata trtn / diff=all notest;

test trtn;

ods output rmst =output rmstdiff = output1;

run;

**Forest Plot in R:**

Please refer to code provided for Supplementary Figure 3

**Plain language summary**

Thymidine kinase 2 deficiency (TK2d) is a very rare and progressive disease caused by changes in genes that are passed down from parents. TK2d reduces the ability of mitochondria (the ‘energy hubs’ of the cell) to produce energy. In people with TK2d, muscles become weaker over time, leading to problems with walking, eating and breathing, and a higher risk of death at an early age.

Previous supportive care strategies did not change how the disease progressed. Pyrimidine nucleos(t)ides^a^ (‘building blocks’ for the body’s genetic material), including doxecitine and doxribtimine, have been studied as potential therapies for TK2d. Doxecitine and doxribtimine is the first approved treatment for patients with TK2d with symptoms starting at or before the age of 12 years in the USA and the European Union, and it works by targeting the root cause of TK2d. Here, data from published scientific reports and medical records were grouped to assess how well pyrimidine nucleos(t)ides therapy works and its safety in patients with TK2d. We compared data from 104 patients who received therapy with 114 patients who did not. Outcomes were looked at in two groups based on the age at which symptoms started: before or after the age of 12 years (reflecting that patients with earlier symptoms tend to have a faster disease progression).

Most patients (approximately 80% of those studied in both the treated and untreated groups) showed symptoms at or before the age of 12 years. For these individuals, a statistical calculation over 30 years of life predicted longer survival time from the start of symptoms with treatment than without (29.2 years vs 14.4 years). The predicted increase in survival time with treatment was smaller for patients with symptoms starting after the age of 12 years (27.2 years vs 24.8 years).

Following treatment, patients were less likely to lose physical motor abilities like walking or standing, and were more likely to regain physical movement abilities they may have previously lost. This was more noticeable in patients with symptoms starting at or before the age of 12 years than in patients with first symptoms after this age. After starting treatment, some patients were able to reduce their use of medical equipment to help with breathing and feeding. Diarrhoea was reported in 86% of patients, but was generally mild or moderate and did not lead to patients stopping treatment in most cases. No serious safety issues were identified.

This study shows that pyrimidine nucleos(t)ide therapy is well tolerated and improves life expectancy and movement-related abilities in patients with TK2d, especially in those who develop symptoms when younger than 12 years of age.

^a^Both pyrimidine nucleoside and nucleotides have been studied as potential therapies for TK2d. Previous studies used nucleotides and ongoing studies are focused on nucleosides.
